# Structural multiplicity in a solvated hydrate of the anti­retroviral protease inhibitor Lopinavir

**DOI:** 10.1107/S2056989024004158

**Published:** 2024-10-24

**Authors:** Tebogo M. L. Mokoto, Andreas Lemmerer, Yasien Sayed, Mark G. Smith

**Affiliations:** a Protein Structure-Function Research Unit, School of Molecular and Cell Biology, University of the Witwatersrand, Johannesburg, Gauteng, South Africa; b Molecular Sciences Institute, School of Chemistry, University of the Witwatersrand, Johannesburg, Gauteng, South Africa; c University of South Africa, Chemistry Department, Unisa Science Campus, 28 Pioneer Avenue, Florida, Roodepoort, Gauteng, South Africa; Universidade de Sâo Paulo, Brazil

**Keywords:** lopinavir, protease inhibitors, crystal structure, heterosolvate, solvate, hydrate, ethyl­ene glycol

## Abstract

Lopinavir is a potent protease inhibitor that is used as a first-line pharmaceutical drug for the treatment of HIV. The multi-component solvated Lopinavir crystal, systematic name (2*S*)-*N*-[(2*S*,4*S*,5*S*)-5-[2-(2,6-di­methyl­phen­oxy)acetamido]-4-hy­droxy-1,6-di­phenyl­hexan-2-yl]-3-methyl-2-(2-oxo-1,3-diazinan-1-yl)butanamide–ethane-1,2-diol–water (8/3/7) 8C_37_H_48_N_4_O_5_·3C_2_H_6_O_2_·7H_2_O, was prepared using evaporative methods. The crystalline material obtained from this experimental synthesis was characterized and elucidated by single-crystal X-ray diffraction (SC-XRD). The crystal structure is unusual in that the unit cell contains 18 mol­ecules. The stoichiometric ratio of this crystal is eight Lopinavir mol­ecules [8(C_37_H_48_N_4_O_5_)], three ethane-1,2-diol mol­ecules [3(C_2_H_6_O_2_)] and seven water mol­ecules [7(H_2_O)]. The crystal packing features both bi- and trifurcated hydrogen bonds between atoms.

## Abstract 2

The multi-component solvated Lopinavir crystal was prepared using evaporative methods. The crystal structure is unusual in that the unit cell contains 18 mol­ecules. The stoichiometric ratio of this crystal is eight Lopinavir mol­ecules, three ethane-1,2-diol mol­ecules and seven water mol­ecules.

## 1. Chemical context

Lopinavir is a protease inhibitor developed from ritonavir (Cvetkovic & Goa, 2003 ▸). It is a highly potent and selective inhibitor of the HIV type-1 protease. Currently, it is available in tablet form and is co-administered with ritonavir. Solvated and hydrated pharmaceutical solids have become a significant area of inter­est lately. This field covers both crystal engineering and the development of efficient pharmaceutical medications. These multi-component crystals are defined by having two or more components (ions, co-formers or solvates) within their crystal structure. During crystallization, it is not uncommon that crystals have both solvents and water incorporating simultaneously in the crystal structure. Solvents in crystallizing systems can affect inter­molecular inter­actions, this in turn changes the inter­nal enthalpy, energy and influences the degree of crystalline disorder and causes entropy changes (Healy *et al.*, 2017 ▸). Hydrates are common since water mol­ecules are readily incorporated into a crystal structure due to their size and multidirectional hydrogen bonds (Gillon *et al.*, 2003 ▸). Solvated hydrated crystals contain one or more solvents as well as water mol­ecules within the crystal structure and occur less frequently. Research on these structures can be deemed advantageous in the understanding of the optimum crystallization process and storage of active pharmaceutical ingredients (Li *et al.*, 2022 ▸).

Solvents and counter-ions are often safely incorporated into pharmaceutical drugs, and these solvents are usually from the ‘Generally Recognised As Safe’ (GRAS) list of solvents (Grothe *et al.*, 2016 ▸). In this study, however, the purpose was simply to understand the structure of Lopinavir crystals. Due to the difficulty in the crystallization process of Lopinavir, a wide range of solvents (both toxic and GRAS solvents) were used for crystallization purposes. Crystallization from ethyl­ene glycol produced a totally unexpected and unusual crystal structure consisting of 18 mol­ecules, namely: Lopinavir, ethane-1,2-diol and water in a ratio of 8:3:7, respectively.
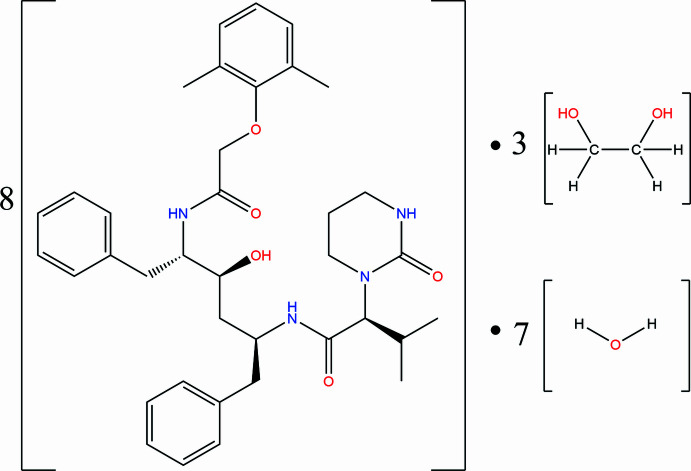


## 2. Structural commentary

**Figure 1:**
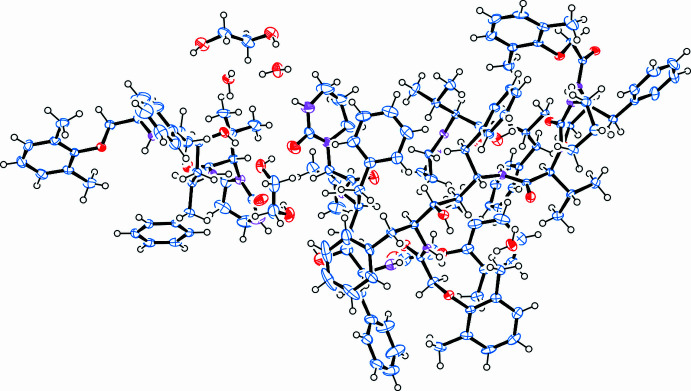
The asymmetric unit of the solvated hydrate of Lopinavir at 50% ellipsoid probability.

**Figure 2:**
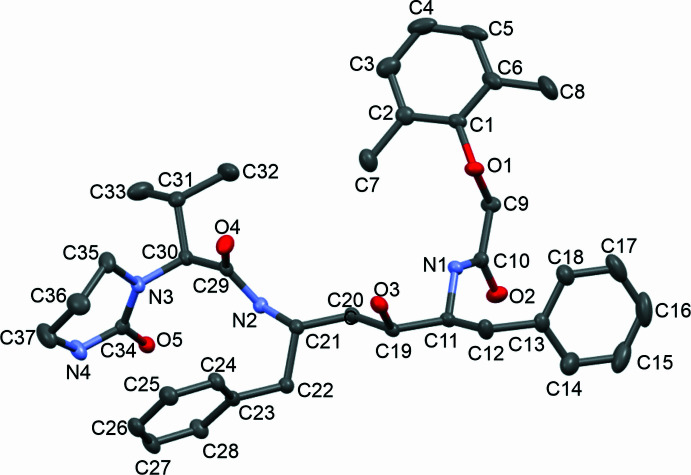
Generalized numbering scheme of the Lopinavir mol­ecules.

**Figure 3:**
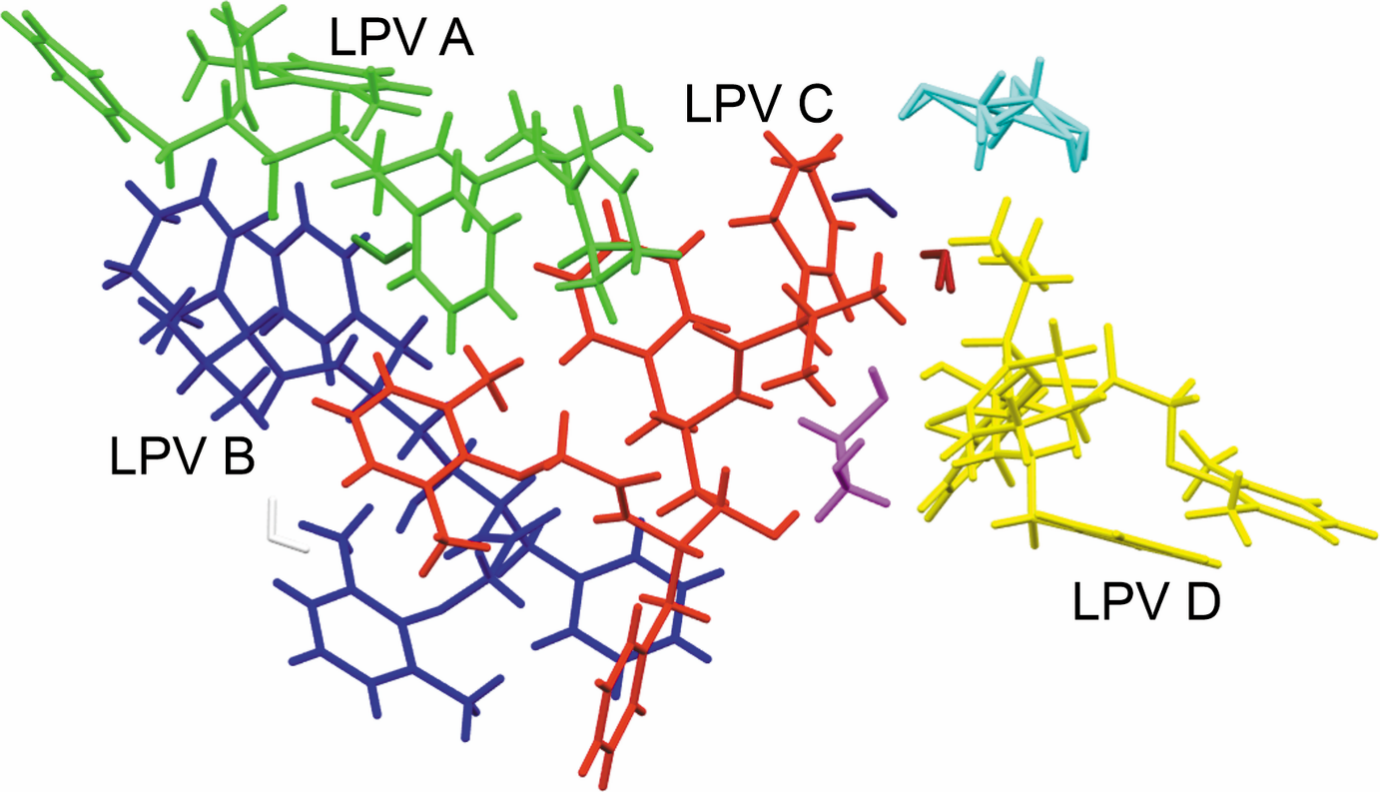
Asymmetric unit of the multi-component Lopinavir solvate.

**Figure 4:**
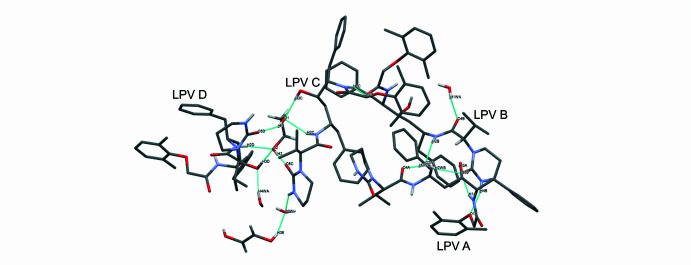
Contents of the asymmetric unit shown by symmetry equivalence, including the naming scheme of the four Lopivavir mol­ecules, LPV A, LPV B, LPV C and LPV D.

The multicomponent solvated structure crystallized in the *C*2 monoclinic space group. The asymmetric unit (Fig. 1 ▸) of this crystal features four Lopinavir mol­ecules (with generalized numbering scheme shown in Fig. 2 ▸), 1.5 ethane-1,2-diol and 3.5 water mol­ecules (Fig. 3 ▸). One of the ethane-1,2-diol mol­ecules is disordered about the twofold axis of the *C*2 space group. The crystal structure is held together by extensive hydrogen bonding (shown in Fig. 4 ▸), and all bond lengths and angles are as expected. The four Lopinavir mol­ecules (LPV) will be referred to as LPV A, LPV B, LPV C and LPV D, respectively (see Fig. 3 ▸ for the assignment of LPV mol­ecules).

**Figure 5:**
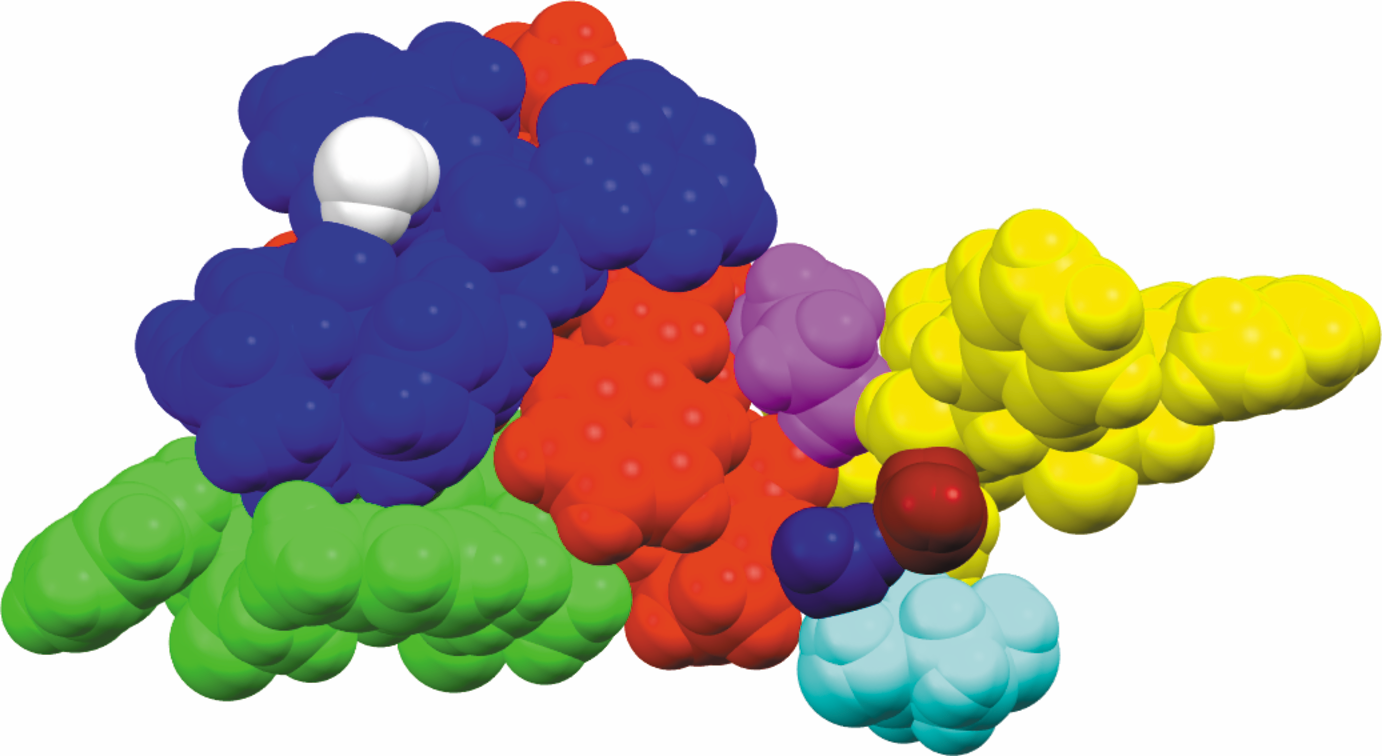
Apart from one water mol­ecule that is encapsulated between two of the Lopinavir mol­ecules, all the remaining water mol­ecules are found on the outside of the shell created by the four LPV mol­ecules.

The first two of the LPV mol­ecules (LPV A and LPV B) inter­act with one another by means of two bifurcated hydrogen bonds from the O5*B* acceptor atom, namely O3*A*—H3*AA*⋯O5*B* and N1*A*—H1*A*⋯O5*B*. The mol­ecule LPV A is also connected to LPV B at a second site *via* a water bridge forming a chain O4*A*⋯H2*WA*—O2*W*—H2*WB*⋯O5*B*, with the expected hydrogen-bond lengths ranging from 1.9–2.1 Å (Jeffrey, 1997 ▸). The above-mentioned water mol­ecule is encapsulated between the LPV A, LPV B and LPV C mol­ecules. However, the remainder of the water mol­ecules within the unit cell are found on the outside of the shell created by the four LPV mol­ecules (Fig. 5 ▸). The LPV B mol­ecule inter­acts with a second water mol­ecule *via* its carbonyl group to form a C29—O4*B*⋯H1*WA* bond, with a bond length of 1.96 Å.

LPV B connects to LPV C *via* a single hydrogen bond N1*C*—H1*C*⋯O2*B*. LPV C additionally bonds to a water mol­ecule by a pyrimidinone moiety *via* N4*C*—H4*C*⋯O3*W*. The disordered ethane-1,2-diol mol­ecule mentioned above bonds to a water mol­ecule by O3—H3*E*⋯O3*W*. In the asymmetric unit, both LPV C and LPV D bond to the same ethane-1,2-diol mol­ecule: LPV C bonds to the OH moiety on one side of the 1,2-ethane­diol mol­ecule (*via* the bifurcated O1⋯H2*C* and O1⋯H3*CA* hydrogen bonds while the LPV D mol­ecule bonds to the second hydroxyl group on the 1,2-ethane­diol mol­ecule.

Inter­estingly, while LPV A, LPV B and LPV C connect directly to each other, LPV D is not directly connected to any other LPV mol­ecule, with only the 1,2-ethane­diol mol­ecule as a bridge between itself and LPV C. LPV D has an additional bond to a water mol­ecule, namely O4*W*—H4*WB*⋯O3*D*.

**Figure 6:**
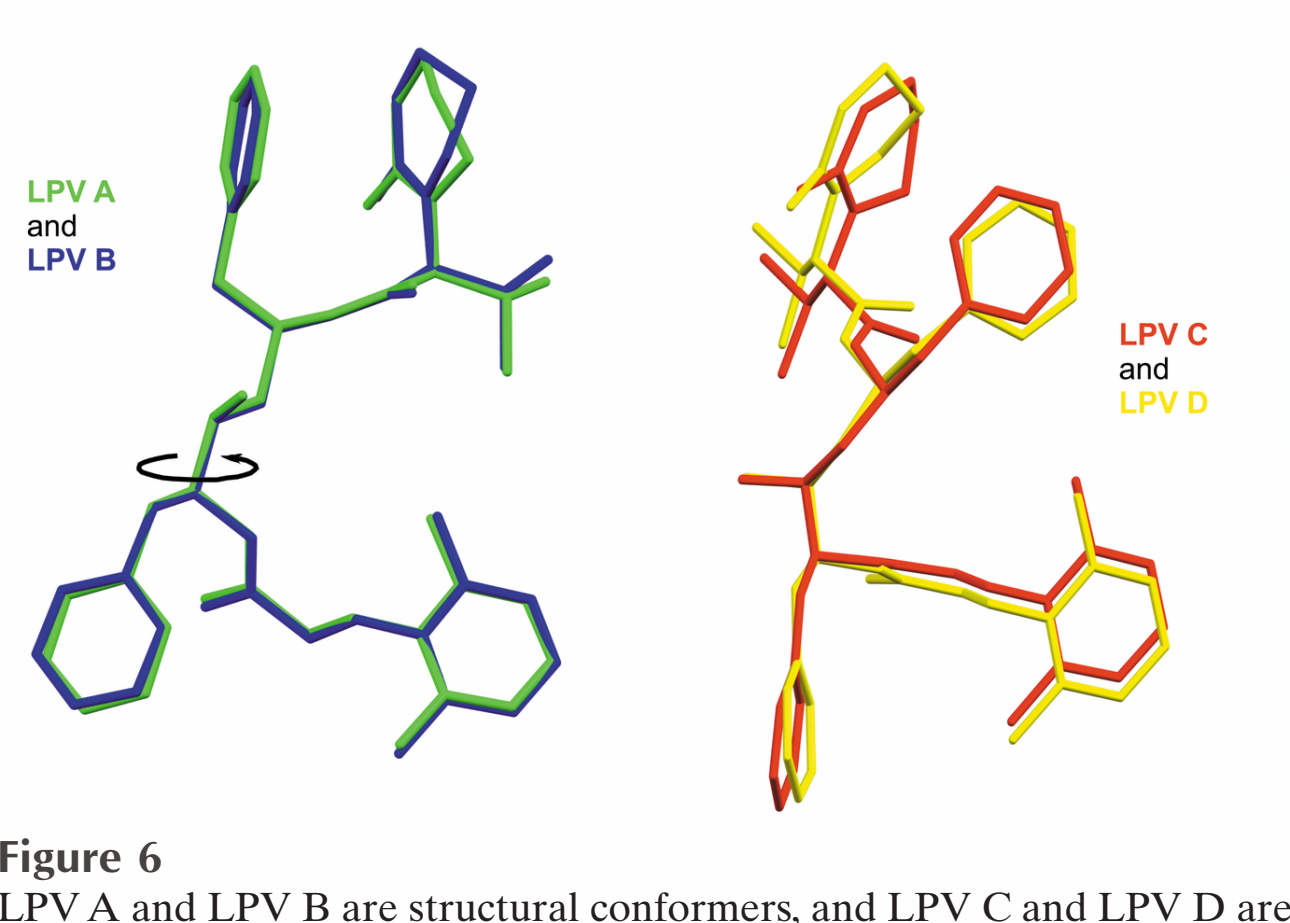
LPV A and LPV B are structural conformers, and LPV C and LPV D are structural conformers (within a small margin of error). However, LPV C and LPV D are rotamers with respect to LPV A and LPV B, with a rotation of 115° around C11—C19.

The Lopinavir mol­ecules in the crystal adopt one of two different conformations (Fig. 6 ▸). LPV A and LPV B are structural conformers, and LPV C and LPV D are structural conformers (within a small margin of error). However, LPV C and LPV D are rotamers with respect to LPV A and LPV B, with a rotation of 115° around C11—C19. These two conformations are similar to those found in host–guest complex of Lopinavir published by Mokoto *et al.* (2024 ▸).

## 3. Supra­molecular features

In the solvated hydrate crystal structure, N2*A*—H2*A*⋯O1*W*^i^ (*x*, *y* + 1, *z*), O1*W*—H1*WB*⋯O5*A*^ii^ (*x*, *y* − 1, *z*), O3*W*—H3*WA*⋯O2*D*^v^ and O4*W*—H4*WA*⋯O3*D*^v^ (−*x* + 1, *y*, −*z* + 2) are hydrogen-bonding inter­actions between amine groups on the LPV mol­ecules attached to several water mol­ecules within the crystal structure (Table 1 ▸). N4—H4*A*⋯O1^i^ hydrogen bonding on the pyrimidinone aromatic ring of the LPV mol­ecule, which is then respectively bound to another LPV mol­ecule. Within the crystalline structure there are several LPV–LPV hydrogen bonds between N1*B*—H1*B*⋯O5*B*^ii^ with a 2.2Å bond length, O3*B*—H3*B*⋯O5*A*^ii^ with a 1.94 Å bond length, N1*D*—H1*D*⋯O2*A*^iii^ (*x* − 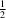, *y* − 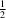, *z*) with a 2.23 Å bond length and N4*D*—H4*D*⋯O2*C*^iv^ (−*x* + 1, *y*, −*z* + 1) with a 2.18 Å bond length, respectively. The packing unit of the crystal shows a centred 180° rotation about the ethane-1,2-diol mol­ecule.

## 4. Database survey

ConQuest (Bruno *et al.*, 2002 ▸), Version 2022.1.0 of the CSD (Groom *et al.*, 2016 ▸) was used for the database survey. To date, no crystal structure of Lopinavir has been published. As of December 2023, a search revealed that there are 73 compounds with ethane-1,2-diol solvates. There are approximately 130 structures of multicomponent solvates on the Cambridge Structural Database (CSD) to date; these include structures that are made up of five different components within the unit cell (Görbitz & Hersleth 2000 ▸).

## 5. Synthesis and crystallization

All reagents were commercially sourced and used without further purification. 0.010 g of Lopinavir (0.0159 mmol) was dissolved in 3 mL of ethyl­ene glycol (ethane-1,2-diol) in a polytop vial at ambient temperature. A 2 mm hole had been made in the lid of the polytop vial using a soldering iron. This was to ensure slow evaporation rather than leaving the complete vial open. The vial was allowed to stand in a dark cabinet in a laboratory with temperatures fluctuating between 283 and 288 K. Colourless blocks were formed after four months and were harvested from the bottom of the vial.

## 6. Refinement

Crystal data, data collection and structure refinement details are summarized in Table 2 ▸. The crystal structure was solved by direct methods using *SHELXT*. Non-hydrogen atoms were first refined isotropically followed by anisotropic refinement by full matrix least-squares calculations based on *F*^2^ using *SHELXL*. Hydrogen atoms attached to carbons were first located in the difference map, then positioned geometrically and allowed to ride on their respective parent atoms, with thermal displacement parameters 1.2 times of the parent C atom. The coordinates and isotropic displacement parameters of the hydrogen atoms attached to O and N atoms that are involved in hydrogen-bonding inter­actions were also refined using riding models.

## Supplementary Material

Crystal structure: contains datablock(s) global, I. DOI: 10.1107/S2056989024004158/ex2081sup1.cif

Structure factors: contains datablock(s) I. DOI: 10.1107/S2056989024004158/ex2081Isup2.hkl

Supporting information file. DOI: 10.1107/S2056989024004158/ex2081Isup3.cml

CCDC reference: 2306134

Additional supporting information:  crystallographic information; 3D view; checkCIF report

## Figures and Tables

**Table 1:** Hydrogen-bond geometry (Å, °)

*D*—H⋯*A*	*D*—H	H⋯*A*	*D*⋯*A*	*D*—H⋯*A*
N1*A*—H1*A*⋯O5*B*	0.88	2.18	2.997 (2)	154
N2*A*—H2*A*⋯O1*W*^i^	0.88	2.06	2.906 (3)	160
N4*A*—H4*A*⋯O1*B*^i^	0.88	2.08	2.933 (3)	163
O3*A*—H3*A*⋯O5*B*	0.84	1.91	2.750 (2)	173
N1*B*—H1*B*⋯O5*A*^ii^	0.88	2.2	3.005 (2)	152
N2*B*—H2*B*⋯O2*W*	0.88	2.05	2.886 (3)	159
N4*B*—H4*B*⋯O1*A*	0.88	2.08	2.932 (3)	164
O3*B*—H3*B*⋯O5*A*^ii^	0.84	1.94	2.771 (2)	170
N1*C*—H1*C*⋯O2*B*	0.88	2.21	3.027 (3)	155
N2*C*—H2*C*⋯O1	0.88	2.18	2.978 (3)	151
N4*C*—H4*C*⋯O3*W*	0.88	2.19	2.983 (3)	150
O3*C*—H3*C*⋯O1	0.81	2.08	2.869 (4)	165
N1*D*—H1*D*⋯O2*A*^iii^	0.88	2.23	3.058 (3)	156
N2*D*—H2*D*⋯O2	0.88	2.13	2.973 (3)	159
N4*D*—H4*D*⋯O2*C*^iv^	0.88	2.18	2.825 (3)	130
O3*D*—H3*D*⋯O2	0.84	1.89	2.730 (3)	178
O1—H1⋯O5*D*	0.84	1.85	2.606 (3)	149
O2—H2⋯O5*C*	0.84	1.74	2.581 (3)	174
O3—H3*O*⋯O3*W*	0.84	2.03	2.856 (12)	167
O1*W*—H1*WB*⋯O5*A*^ii^	0.87	2.05	2.861 (2)	154
O1*W*—H1*WA*⋯O4*B*	0.87	1.96	2.818 (2)	169
O2*W*—H2*WA*⋯O4*A*	0.87	1.95	2.810 (2)	171
O2*W*—H2*WB*⋯O5*B*	0.87	2.08	2.907 (2)	158
O3*W*—H3*WA*⋯O2*D*^v^	0.87	1.9	2.769 (3)	173
O3*W*—H3*WB*⋯O4*W*	0.87	1.93	2.798 (2)	172
O4*W*—H4*WB*⋯O3*D*	0.87	1.89	2.736 (3)	164
O4*W*—H4*WA*⋯O3*D*^v^	0.87	1.89	2.736 (3)	164
O1—H1⋯O5*D*	0.84	1.85	2.606 (3)	149
O2—H2⋯O5*C*	0.84	1.74	2.581 (3)	174
O3—H3*O*⋯O3*W*	0.84	2.03	2.856 (12)	167

Symmetry codes: (i) 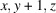; (ii) 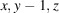; (iii) 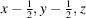; (iv) 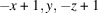; (v) 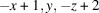.

**Table 2:** Experimental details

Crystal data	
Chemical formula	8C_37_H_48_N_4_O_5_·3C_2_H_6_O_2_·7H_2_O
*M*_r_	5342.64
Crystal system, space group	Monoclinic, *C*2
Temperature (K)	123
*a*, *b*, *c* (Å)	46.5945 (13), 13.9309 (4), 23.4225 (7)
β (°)	104.053 (1)
*V* (Å^3^)	14748.6 (7)
*Z*	2
Radiation type	Cu *K*α
μ (mm^−1^)	0.67
Crystal size (mm)	0.43 × 0.20 × 0.03

Data collection	
Diffractometer	Bruker D8 Venture Photon CCD area detector
Absorption correction	Multi-scan (*SADABS*; Krause *et al.*, 2015 ▸)
*T*_min_, *T*_max_	0.639, 0.754
No. of measured, independent and observed [*I* > 2σ(*I*)] reflections	135449, 27271, 25438
*R*_int_	0.039
(sin θ/λ)_max_ (Å^−1^)	0.621

Refinement	
*R*[*F*^2^ > 2σ(*F*^2^)], *wR*(*F*^2^), *S*	0.038, 0.099, 1.03
No. of reflections	27271
No. of parameters	1783
No. of restraints	3
H-atom treatment	H-atom parameters constrained
Δρ_max_, Δρ_min_ (e Å^−3^)	0.46, −0.36
Absolute structure	Flack *x* determined using 10831 quotients [(*I*^+^)−(*I*^−^)]/[(*I*^+^)+(*I*^−^)] (Parsons *et al.*, 2013 ▸)
Absolute structure parameter	0.01 (4)

Computer programs: *APEX3*, *SAINT-Plus* and *XPREP* (Bruker 2016 ▸), *SHELXT2014* (Sheldrick, 2015*a* ▸), *SHELXL2019/2* (Sheldrick, 2015*b* ▸) and *ORTEP-3 for Windows* and *WinGX* publication routines (Farrugia, 2012 ▸).

## supplementary crystallographic information

### (2S)-N-[(2S,4S,5S)-5-[2-(2,6-Dimethylphenoxy)acetamido]-4-hydroxy-1,6-diphenylhexan-2-yl]-3-methyl-2-(2-oxo-1,3-diazinan-1-yl)butanamide–ethane-1,2-diol–water (8/2/1/7) Crystal data

**Table:**

8C_37_H_48_N_4_O_5_·3C_2_H_6_O_2_·7H_2_O	*F*(000) = 5752
*M**_r_* = 5342.64	*D*_x_ = 1.203 Mg m^−^^3^
Monoclinic, *C*2	Cu *K*α radiation, λ = 1.54178 Å
Hall symbol: C 2y	Cell parameters from 9081 reflections
*a* = 46.5945 (13) Å	θ = 3.1–71.8°
*b* = 13.9309 (4) Å	µ = 0.67 mm^−^^1^
*c* = 23.4225 (7) Å	*T* = 123 K
β = 104.053 (1)°	Plate, colourless
*V* = 14748.6 (7) Å^3^	0.43 × 0.20 × 0.03 mm
*Z* = 2	

### (2S)-N-[(2S,4S,5S)-5-[2-(2,6-Dimethylphenoxy)acetamido]-4-hydroxy-1,6-diphenylhexan-2-yl]-3-methyl-2-(2-oxo-1,3-diazinan-1-yl)butanamide–ethane-1,2-diol–water (8/2/1/7) Data collection

**Table:**

Bruker D8 Venture Photon CCD area detector diffractometer	25438 reflections with *I* > 2σ(*I*)
ω scans	*R*_int_ = 0.039
Absorption correction: multi-scan (SADABS; Krause *et al.*, 2015)	θ_max_ = 73.1°, θ_min_ = 1.9°
*T*_min_ = 0.639, *T*_max_ = 0.754	*h* = −57→57
135449 measured reflections	*k* = −16→15
27271 independent reflections	*l* = −29→28

### (2S)-N-[(2S,4S,5S)-5-[2-(2,6-Dimethylphenoxy)acetamido]-4-hydroxy-1,6-diphenylhexan-2-yl]-3-methyl-2-(2-oxo-1,3-diazinan-1-yl)butanamide–ethane-1,2-diol–water (8/2/1/7) Refinement

**Table:**

Refinement on *F*^2^	Secondary atom site location: dual
Least-squares matrix: full	Hydrogen site location: mixed
*R*[*F*^2^ > 2σ(*F*^2^)] = 0.038	H-atom parameters constrained
*wR*(*F*^2^) = 0.099	*w* = 1/[σ^2^(*F*_o_^2^) + (0.0546*P*)^2^ + 7.469*P*] where *P* = (*F*_o_^2^ + 2*F*_c_^2^)/3
*S* = 1.03	(Δ/σ)_max_ = 0.001
27271 reflections	Δρ_max_ = 0.46 e Å^−^^3^
1783 parameters	Δρ_min_ = −0.36 e Å^−^^3^
3 restraints	Absolute structure: Flack *x* determined using 10831 quotients [(*I*^+^)-(*I*^-^)]/[(*I*^+^)+(*I*^-^)] (Parsons *et al.*, 2013)
0 constraints	Absolute structure parameter: 0.01 (4)
Primary atom site location: dual	

### (2S)-N-[(2S,4S,5S)-5-[2-(2,6-Dimethylphenoxy)acetamido]-4-hydroxy-1,6-diphenylhexan-2-yl]-3-methyl-2-(2-oxo-1,3-diazinan-1-yl)butanamide–ethane-1,2-diol–water (8/2/1/7) Special details

**Table:**

Geometry. All esds (except the esd in the dihedral angle between two l.s. planes) are estimated using the full covariance matrix. The cell esds are taken into account individually in the estimation of esds in distances, angles and torsion angles; correlations between esds in cell parameters are only used when they are defined by crystal symmetry. An approximate (isotropic) treatment of cell esds is used for estimating esds involving l.s. planes.

### (2S)-N-[(2S,4S,5S)-5-[2-(2,6-Dimethylphenoxy)acetamido]-4-hydroxy-1,6-diphenylhexan-2-yl]-3-methyl-2-(2-oxo-1,3-diazinan-1-yl)butanamide–ethane-1,2-diol–water (8/2/1/7) Fractional atomic coordinates and isotropic or equivalent isotropic displacement parameters (Å^2^)

**Table:**

	*x*	*y*	*z*	*U*_iso_*/*U*_eq_	Occ. (<1)
C1A	0.83757 (5)	0.61700 (16)	0.98246 (9)	0.0188 (5)	
C2A	0.80791 (5)	0.64260 (17)	0.97146 (11)	0.0229 (5)	
C3A	0.79358 (7)	0.6287 (2)	1.01622 (12)	0.0333 (6)	
H3AA	0.773338	0.645991	1.010023	0.04*	
C4A	0.80824 (7)	0.5901 (2)	1.06966 (12)	0.0363 (7)	
H4AA	0.798099	0.581202	1.099889	0.044*	
C5A	0.83759 (7)	0.5646 (2)	1.07910 (11)	0.0353 (7)	
H5A	0.847475	0.537796	1.115916	0.042*	
C6A	0.85303 (6)	0.57747 (18)	1.03577 (10)	0.0260 (5)	
C7A	0.79190 (6)	0.6825 (2)	0.91271 (12)	0.0315 (6)	
H7AA	0.80042	0.744849	0.906578	0.047*	
H7AB	0.770899	0.690517	0.911774	0.047*	
H7AC	0.793959	0.638115	0.881482	0.047*	
C8A	0.88480 (7)	0.5468 (3)	1.04490 (14)	0.0459 (8)	
H8AA	0.885764	0.487599	1.022812	0.069*	
H8AB	0.893317	0.535296	1.086885	0.069*	
H8AC	0.896012	0.597427	1.030953	0.069*	
C9A	0.86577 (5)	0.71699 (17)	0.93424 (10)	0.0223 (5)	
H9AA	0.886375	0.714874	0.958468	0.027*	
H9AB	0.855011	0.766762	0.950929	0.027*	
C10A	0.86593 (5)	0.74391 (16)	0.87188 (10)	0.0175 (4)	
C11A	0.85274 (5)	0.69944 (17)	0.76748 (9)	0.0180 (5)	
H11A	0.860529	0.765493	0.763941	0.022*	
C12A	0.87436 (5)	0.62770 (19)	0.75032 (10)	0.0245 (5)	
H12A	0.8667	0.561824	0.752389	0.029*	
H12B	0.875051	0.639844	0.709037	0.029*	
C13A	0.90536 (5)	0.63328 (18)	0.78890 (10)	0.0218 (5)	
C14A	0.92593 (6)	0.6950 (2)	0.77532 (14)	0.0351 (6)	
H14A	0.920705	0.733055	0.740688	0.042*	
C15A	0.95430 (6)	0.7019 (2)	0.81201 (17)	0.0476 (8)	
H15A	0.968384	0.743867	0.801948	0.057*	
C16A	0.96192 (7)	0.6486 (3)	0.86245 (16)	0.0489 (9)	
H16A	0.981184	0.654088	0.887641	0.059*	
C17A	0.94166 (7)	0.5869 (2)	0.87666 (13)	0.0444 (8)	
H17A	0.946861	0.550099	0.911802	0.053*	
C18A	0.91351 (6)	0.5786 (2)	0.83940 (11)	0.0292 (6)	
H18A	0.89975	0.534697	0.84885	0.035*	
C19A	0.82205 (5)	0.69263 (16)	0.72481 (9)	0.0171 (4)	
H19A	0.824206	0.718278	0.686195	0.021*	
C20A	0.79889 (5)	0.75547 (16)	0.74332 (9)	0.0176 (4)	
H20A	0.806036	0.822635	0.747448	0.021*	
H20B	0.796702	0.733846	0.7823	0.021*	
C21A	0.76857 (5)	0.75250 (16)	0.69951 (9)	0.0167 (4)	
H21A	0.762549	0.683819	0.692186	0.02*	
C22A	0.76891 (5)	0.79929 (17)	0.64035 (10)	0.0189 (5)	
H22A	0.77384	0.868151	0.647051	0.023*	
H22B	0.784765	0.769215	0.624965	0.023*	
C23A	0.73995 (5)	0.79074 (17)	0.59423 (9)	0.0184 (4)	
C24A	0.72838 (5)	0.70122 (17)	0.57497 (10)	0.0218 (5)	
H24A	0.738205	0.645077	0.592773	0.026*	
C25A	0.70275 (5)	0.69195 (18)	0.53024 (10)	0.0250 (5)	
H25A	0.695187	0.630148	0.5176	0.03*	
C26A	0.68835 (5)	0.77370 (19)	0.50428 (10)	0.0261 (5)	
H26A	0.671048	0.768093	0.473136	0.031*	
C27A	0.69915 (6)	0.86352 (19)	0.52367 (11)	0.0272 (5)	
H27A	0.689045	0.919512	0.506311	0.033*	
C28A	0.72477 (5)	0.87200 (18)	0.56852 (11)	0.0242 (5)	
H28A	0.731989	0.933907	0.581765	0.029*	
C29A	0.72302 (5)	0.75646 (16)	0.73552 (10)	0.0179 (5)	
C30A	0.69987 (5)	0.82259 (16)	0.75096 (10)	0.0177 (5)	
H30A	0.709175	0.886904	0.761577	0.021*	
C31A	0.68861 (5)	0.78582 (18)	0.80346 (10)	0.0219 (5)	
H31A	0.677346	0.72503	0.791425	0.026*	
C32A	0.71484 (6)	0.7634 (2)	0.85499 (11)	0.0364 (7)	
H32A	0.726029	0.709321	0.844636	0.055*	
H32B	0.707606	0.746735	0.889673	0.055*	
H32C	0.727714	0.819869	0.863743	0.055*	
C33A	0.66784 (7)	0.8585 (2)	0.82015 (13)	0.0367 (7)	
H33A	0.678378	0.91917	0.831107	0.055*	
H33B	0.660795	0.834116	0.853561	0.055*	
H33C	0.650936	0.869141	0.786571	0.055*	
C34A	0.67173 (5)	0.91782 (16)	0.66817 (10)	0.0168 (4)	
C35A	0.65534 (5)	0.75202 (17)	0.68014 (11)	0.0256 (5)	
H35A	0.638809	0.756693	0.699754	0.031*	
H35B	0.666067	0.691361	0.692814	0.031*	
C36A	0.64330 (6)	0.75078 (19)	0.61497 (12)	0.0327 (6)	
H36A	0.628274	0.699394	0.604229	0.039*	
H36B	0.659452	0.736842	0.595462	0.039*	
C37A	0.62941 (6)	0.8466 (2)	0.59387 (13)	0.0355 (6)	
H37A	0.625365	0.84988	0.550424	0.043*	
H37B	0.610381	0.853353	0.605205	0.043*	
N1A	0.85176 (4)	0.68550 (13)	0.82891 (8)	0.0161 (4)	
H1A	0.841516	0.637137	0.837861	0.019*	
N2A	0.74653 (4)	0.80093 (13)	0.72413 (8)	0.0165 (4)	
H2A	0.748792	0.862653	0.731906	0.02*	
N3A	0.67560 (4)	0.83400 (13)	0.69800 (8)	0.0182 (4)	
N4A	0.64919 (4)	0.92415 (15)	0.61968 (9)	0.0246 (4)	
H4A	0.646194	0.980363	0.602092	0.03*	
O1A	0.85206 (4)	0.62592 (11)	0.93690 (7)	0.0195 (3)	
O2A	0.87903 (4)	0.81798 (12)	0.86411 (7)	0.0249 (4)	
O3A	0.81164 (3)	0.59616 (11)	0.71429 (7)	0.0205 (3)	
H3A	0.811873	0.569346	0.746517	0.031*	
O4A	0.71937 (4)	0.66856 (12)	0.73258 (8)	0.0255 (4)	
O5A	0.68791 (3)	0.99020 (11)	0.68402 (7)	0.0188 (3)	
C1B	0.66641 (5)	0.10816 (16)	0.51803 (10)	0.0190 (5)	
C2B	0.65027 (6)	0.08022 (17)	0.46225 (11)	0.0257 (5)	
C3B	0.66561 (7)	0.07073 (19)	0.41840 (11)	0.0325 (6)	
H3BB	0.655286	0.050957	0.3801	0.039*	
C4B	0.69552 (7)	0.0895 (2)	0.42959 (12)	0.0338 (6)	
H4BB	0.705653	0.082173	0.399214	0.041*	
C5B	0.71081 (6)	0.11917 (19)	0.48524 (12)	0.0291 (6)	
H5B	0.731349	0.132922	0.492425	0.035*	
C6B	0.69660 (5)	0.12907 (17)	0.53056 (10)	0.0222 (5)	
C7B	0.61751 (6)	0.0624 (2)	0.45018 (13)	0.0397 (7)	
H7BA	0.613312	0.021154	0.481087	0.06*	
H7BB	0.610734	0.030605	0.411931	0.06*	
H7BC	0.607127	0.123652	0.449649	0.06*	
C8B	0.71289 (6)	0.1618 (2)	0.59071 (11)	0.0295 (6)	
H8BA	0.706401	0.226713	0.597802	0.044*	
H8BB	0.734195	0.162267	0.593278	0.044*	
H8BC	0.708703	0.117897	0.620358	0.044*	
C9B	0.63574 (6)	0.19679 (18)	0.56697 (10)	0.0265 (5)	
H9BA	0.615267	0.187648	0.543081	0.032*	
H9BB	0.64463	0.250513	0.54956	0.032*	
C10B	0.63484 (5)	0.22287 (16)	0.62909 (10)	0.0187 (5)	
C11B	0.65023 (5)	0.18467 (17)	0.73412 (9)	0.0186 (5)	
H11B	0.641182	0.249113	0.736729	0.022*	
C12B	0.63036 (5)	0.1094 (2)	0.75345 (11)	0.0259 (5)	
H12C	0.638762	0.044856	0.750292	0.031*	
H12D	0.63073	0.120334	0.79538	0.031*	
C13B	0.59879 (5)	0.11034 (18)	0.71838 (11)	0.0254 (5)	
C14B	0.57765 (7)	0.1638 (2)	0.73689 (17)	0.0457 (8)	
H14B	0.58317	0.199914	0.772277	0.055*	
C15B	0.54854 (8)	0.1652 (3)	0.7043 (3)	0.0734 (14)	
H15B	0.534194	0.201674	0.717544	0.088*	
C16B	0.54040 (8)	0.1138 (3)	0.6528 (2)	0.0753 (15)	
H16B	0.520504	0.115506	0.630116	0.09*	
C17B	0.56093 (8)	0.0604 (3)	0.63423 (16)	0.0617 (11)	
H17B	0.555227	0.02452	0.598796	0.074*	
C18B	0.59021 (6)	0.0581 (2)	0.66684 (12)	0.0363 (6)	
H18B	0.604334	0.020467	0.653634	0.044*	
C19B	0.68114 (5)	0.18490 (16)	0.77646 (10)	0.0181 (4)	
H19B	0.678514	0.210153	0.814839	0.022*	
C20B	0.70289 (5)	0.25254 (17)	0.75737 (10)	0.0184 (5)	
H20C	0.694466	0.318126	0.75272	0.022*	
H20D	0.705608	0.231582	0.718641	0.022*	
C21B	0.73312 (5)	0.25500 (16)	0.80185 (9)	0.0172 (4)	
H21B	0.740228	0.187461	0.809849	0.021*	
C22B	0.73173 (5)	0.30157 (17)	0.86058 (10)	0.0199 (5)	
H22C	0.726205	0.369969	0.853696	0.024*	
H22D	0.71609	0.269656	0.875739	0.024*	
C23B	0.76077 (5)	0.29499 (17)	0.90657 (9)	0.0186 (5)	
C24B	0.77754 (5)	0.37619 (17)	0.92685 (10)	0.0223 (5)	
H24B	0.771114	0.43732	0.910733	0.027*	
C25B	0.80362 (6)	0.36847 (19)	0.97054 (11)	0.0263 (5)	
H25B	0.814721	0.424497	0.984576	0.032*	
C26B	0.81353 (5)	0.2796 (2)	0.99377 (11)	0.0276 (5)	
H26B	0.831385	0.274624	1.023592	0.033*	
C27B	0.79722 (6)	0.19785 (19)	0.97323 (11)	0.0263 (5)	
H27B	0.803918	0.136645	0.988832	0.032*	
C28B	0.77113 (5)	0.20587 (17)	0.92986 (10)	0.0220 (5)	
H28B	0.760124	0.149676	0.915777	0.026*	
C29B	0.77839 (5)	0.26531 (16)	0.76609 (10)	0.0184 (5)	
C30B	0.80244 (5)	0.33373 (16)	0.75599 (10)	0.0176 (4)	
H30B	0.793268	0.398531	0.746691	0.021*	
C31B	0.81445 (5)	0.30198 (17)	0.70340 (10)	0.0220 (5)	
H31B	0.820996	0.233543	0.709029	0.026*	
C32B	0.78935 (6)	0.3099 (2)	0.64801 (11)	0.0365 (7)	
H32D	0.772988	0.268088	0.651756	0.055*	
H32E	0.796498	0.290178	0.613745	0.055*	
H32F	0.782426	0.376497	0.642934	0.055*	
C33B	0.84038 (6)	0.36359 (19)	0.69742 (11)	0.0275 (5)	
H33D	0.834582	0.431364	0.695424	0.041*	
H33E	0.846282	0.345863	0.661421	0.041*	
H33F	0.85703	0.353368	0.73156	0.041*	
C34B	0.83133 (5)	0.42742 (16)	0.83846 (10)	0.0172 (4)	
C35B	0.84141 (6)	0.25483 (18)	0.83696 (12)	0.0275 (5)	
H35C	0.841903	0.208133	0.805358	0.033*	
H35D	0.830729	0.224862	0.864117	0.033*	
C36B	0.87264 (6)	0.27841 (19)	0.87013 (12)	0.0303 (6)	
H36C	0.882445	0.219853	0.889537	0.036*	
H36D	0.884026	0.301805	0.842317	0.036*	
C37B	0.87222 (6)	0.35395 (19)	0.91552 (11)	0.0265 (5)	
H37C	0.864437	0.32658	0.947754	0.032*	
H37D	0.89256	0.377475	0.932455	0.032*	
N1B	0.65130 (4)	0.16992 (13)	0.67283 (8)	0.0165 (4)	
H1B	0.66301	0.125292	0.664465	0.02*	
N2B	0.75487 (4)	0.30756 (13)	0.77832 (8)	0.0176 (4)	
H2B	0.752345	0.369595	0.771947	0.021*	
N3B	0.82559 (4)	0.34188 (14)	0.81104 (8)	0.0184 (4)	
N4B	0.85350 (4)	0.43287 (14)	0.88779 (9)	0.0225 (4)	
H4B	0.856932	0.489497	0.904725	0.027*	
O1B	0.65233 (3)	0.11160 (11)	0.56449 (7)	0.0197 (3)	
O2B	0.61939 (4)	0.29131 (12)	0.63620 (7)	0.0250 (4)	
O3B	0.69333 (4)	0.09092 (11)	0.78812 (7)	0.0210 (3)	
H3B	0.693574	0.06379	0.756208	0.031*	
O4B	0.78157 (4)	0.17741 (12)	0.76419 (8)	0.0278 (4)	
O5B	0.81706 (3)	0.50268 (11)	0.81979 (7)	0.0201 (3)	
C1C	0.66967 (6)	0.42174 (18)	0.54508 (11)	0.0275 (5)	
C2C	0.67956 (6)	0.38950 (17)	0.49669 (11)	0.0271 (5)	
C3C	0.70986 (6)	0.3935 (2)	0.50066 (12)	0.0333 (6)	
H3CC	0.71724	0.373553	0.468152	0.04*	
C4C	0.72929 (7)	0.4261 (2)	0.55106 (13)	0.0399 (7)	
H4CC	0.74984	0.42958	0.552681	0.048*	
C5C	0.71909 (7)	0.4536 (2)	0.59912 (13)	0.0395 (7)	
H5C	0.732803	0.473749	0.63404	0.047*	
C6C	0.68900 (7)	0.45231 (18)	0.59730 (12)	0.0327 (6)	
C7C	0.65882 (6)	0.3491 (2)	0.44244 (11)	0.0306 (6)	
H7CA	0.662852	0.2805	0.439228	0.046*	
H7CB	0.661771	0.382684	0.407556	0.046*	
H7CC	0.63833	0.357731	0.445242	0.046*	
C8C	0.67720 (8)	0.4817 (2)	0.64893 (13)	0.0474 (8)	
H8CA	0.665299	0.540124	0.639063	0.071*	
H8CB	0.693736	0.494143	0.68295	0.071*	
H8CC	0.664853	0.430099	0.658475	0.071*	
C9C	0.62329 (7)	0.4999 (2)	0.51707 (15)	0.0436 (7)	
H9CA	0.626575	0.510282	0.477327	0.052*	
H9CB	0.630601	0.557185	0.54121	0.052*	
C10C	0.59084 (8)	0.4881 (2)	0.51238 (13)	0.0437 (7)	
C11C	0.55105 (6)	0.3909 (2)	0.53315 (12)	0.0360 (7)	
H11C	0.53937	0.420922	0.495937	0.043*	
C12C	0.54508 (7)	0.2834 (2)	0.52903 (12)	0.0378 (7)	
H12E	0.559696	0.251484	0.5612	0.045*	
H12F	0.525252	0.271904	0.536068	0.045*	
C13C	0.54621 (6)	0.2355 (2)	0.47168 (13)	0.0398 (7)	
C14C	0.55169 (9)	0.2834 (3)	0.42477 (14)	0.0574 (10)	
H14C	0.555381	0.35055	0.427664	0.069*	
C15C	0.55196 (10)	0.2355 (3)	0.37230 (16)	0.0694 (13)	
H15C	0.556057	0.270121	0.340251	0.083*	
C16C	0.54631 (8)	0.1384 (3)	0.36695 (16)	0.0606 (11)	
H16C	0.546309	0.106024	0.331249	0.073*	
C17C	0.54080 (7)	0.0898 (3)	0.41290 (18)	0.0573 (10)	
H17C	0.537109	0.022689	0.409691	0.069*	
C18C	0.54048 (7)	0.1374 (3)	0.46513 (16)	0.0482 (8)	
H18C	0.536269	0.102208	0.496889	0.058*	
C19C	0.54188 (6)	0.4395 (2)	0.58438 (13)	0.0409 (7)	
H19C	0.546094	0.5097	0.583088	0.049*	
C19D	0.44575 (5)	0.51720 (19)	0.90024 (11)	0.0258 (5)	
H19D	0.436383	0.58221	0.896756	0.031*	
C20C	0.55800 (6)	0.4009 (2)	0.64506 (12)	0.0310 (6)	
H20E	0.543113	0.376423	0.665244	0.037*	
H20F	0.570581	0.346125	0.639523	0.037*	
C21C	0.57727 (5)	0.47486 (19)	0.68459 (11)	0.0268 (5)	
H21C	0.593303	0.495953	0.665735	0.032*	
C22C	0.59146 (6)	0.4281 (2)	0.74437 (11)	0.0293 (6)	
H22E	0.575484	0.406618	0.762567	0.035*	
H22F	0.602492	0.370375	0.737307	0.035*	
C23C	0.61215 (6)	0.49186 (19)	0.78733 (11)	0.0279 (5)	
C24C	0.64217 (6)	0.4945 (2)	0.78828 (12)	0.0331 (6)	
H24C	0.649486	0.456306	0.761333	0.04*	
C25C	0.66150 (7)	0.5525 (2)	0.82831 (14)	0.0411 (7)	
H25C	0.681935	0.553505	0.828743	0.049*	
C26C	0.65110 (8)	0.6085 (2)	0.86726 (13)	0.0435 (8)	
H26C	0.664341	0.647973	0.89463	0.052*	
C27C	0.62139 (8)	0.6072 (2)	0.86658 (12)	0.0407 (7)	
H27C	0.614184	0.646226	0.893324	0.049*	
C28C	0.60208 (7)	0.5493 (2)	0.82698 (12)	0.0351 (6)	
H28C	0.581679	0.548682	0.826894	0.042*	
C29C	0.56685 (6)	0.6496 (2)	0.68410 (12)	0.0305 (6)	
C30C	0.54336 (6)	0.7236 (2)	0.68970 (12)	0.0309 (6)	
H30C	0.524798	0.687738	0.689947	0.037*	
C31C	0.53645 (7)	0.7917 (2)	0.63659 (14)	0.0407 (7)	
H31C	0.554777	0.82851	0.635794	0.049*	
C32C	0.52708 (9)	0.7336 (3)	0.57972 (15)	0.0642 (11)	
H32G	0.543225	0.690575	0.576272	0.096*	
H32H	0.522538	0.777318	0.545959	0.096*	
H32I	0.509489	0.695535	0.580499	0.096*	
C33C	0.51236 (7)	0.8625 (2)	0.64175 (18)	0.0538 (9)	
H33G	0.494265	0.827314	0.64269	0.081*	
H33H	0.508399	0.905945	0.607838	0.081*	
H33I	0.51889	0.899808	0.678059	0.081*	
C34C	0.54253 (6)	0.74148 (19)	0.79239 (12)	0.0313 (6)	
C35C	0.57819 (9)	0.8384 (3)	0.75656 (16)	0.0614 (11)	
H35E	0.596622	0.800053	0.764106	0.074*	
H35F	0.577179	0.87724	0.720739	0.074*	
C36C	0.57922 (10)	0.9021 (3)	0.80605 (17)	0.0676 (12)	
H36E	0.598861	0.93353	0.816177	0.081*	
H36F	0.564245	0.953058	0.793189	0.081*	
C37C	0.57427 (11)	0.8592 (3)	0.85840 (18)	0.0756 (14)	
H37E	0.567651	0.90922	0.882404	0.091*	
H37F	0.593081	0.832122	0.881917	0.091*	
N1C	0.58238 (5)	0.41107 (17)	0.53753 (9)	0.0323 (5)	
H1C	0.59588	0.371306	0.557221	0.039*	
N2C	0.55947 (4)	0.55791 (16)	0.69162 (9)	0.0259 (4)	
H2C	0.542689	0.547382	0.701486	0.031*	
N3C	0.55281 (5)	0.77350 (17)	0.74625 (10)	0.0330 (5)	
N4C	0.55208 (6)	0.7835 (2)	0.84491 (11)	0.0472 (7)	
H4C	0.544349	0.763856	0.873613	0.057*	
O1C	0.63957 (4)	0.41781 (13)	0.54297 (8)	0.0308 (4)	
O2C	0.57360 (6)	0.5486 (2)	0.48485 (13)	0.0711 (8)	
O3C	0.51052 (5)	0.4268 (2)	0.57400 (12)	0.0704 (9)	
H3C	0.505873	0.453202	0.601401	0.106*	
O4C	0.59044 (4)	0.67346 (16)	0.67377 (10)	0.0440 (5)	
O5C	0.52437 (5)	0.67566 (17)	0.78589 (10)	0.0523 (6)	
C1D	0.32293 (5)	0.43588 (16)	0.95413 (10)	0.0204 (5)	
C2D	0.30220 (6)	0.46001 (17)	0.90246 (11)	0.0237 (5)	
C3D	0.27249 (6)	0.45574 (18)	0.90330 (12)	0.0299 (6)	
H3DD	0.257769	0.471702	0.868846	0.036*	
C4D	0.26410 (6)	0.4285 (2)	0.95380 (14)	0.0354 (6)	
H4DD	0.243759	0.428214	0.954211	0.042*	
C5D	0.28526 (6)	0.4016 (2)	1.00364 (12)	0.0324 (6)	
H5D	0.279229	0.381309	1.037664	0.039*	
C6D	0.31520 (6)	0.40377 (18)	1.00466 (11)	0.0253 (5)	
C7D	0.31180 (7)	0.4881 (2)	0.84782 (11)	0.0353 (6)	
H7DA	0.314848	0.55773	0.847675	0.053*	
H7DB	0.29646	0.469711	0.812938	0.053*	
H7DC	0.330325	0.45526	0.847285	0.053*	
C8D	0.33821 (7)	0.3701 (2)	1.05816 (11)	0.0326 (6)	
H8DA	0.342466	0.301964	1.053696	0.049*	
H8DB	0.330693	0.378816	1.093427	0.049*	
H8DC	0.35637	0.407576	1.06201	0.049*	
C9D	0.36398 (6)	0.53616 (19)	0.97262 (12)	0.0297 (6)	
H9DA	0.360619	0.550774	1.011856	0.036*	
H9DB	0.353053	0.584199	0.944418	0.036*	
C10D	0.39637 (6)	0.5434 (2)	0.97531 (11)	0.0291 (6)	
C11D	0.44039 (5)	0.46847 (19)	0.95550 (11)	0.0261 (5)	
H11D	0.450779	0.506948	0.990465	0.031*	
C12D	0.45240 (6)	0.3664 (2)	0.96362 (11)	0.0314 (6)	
H12G	0.441738	0.327711	0.92959	0.038*	
H12H	0.473478	0.368154	0.962372	0.038*	
C13D	0.45027 (6)	0.3149 (2)	1.01925 (12)	0.0333 (6)	
C14D	0.44131 (10)	0.3573 (3)	1.06513 (14)	0.0596 (10)	
H14D	0.436396	0.423585	1.06338	0.071*	
C15D	0.43942 (12)	0.3029 (3)	1.11438 (16)	0.0750 (14)	
H15D	0.432589	0.332306	1.145206	0.09*	
C16D	0.44730 (10)	0.2076 (3)	1.11883 (16)	0.0643 (11)	
H16D	0.44708	0.172173	1.153378	0.077*	
C17D	0.45533 (7)	0.1654 (3)	1.07340 (15)	0.0500 (9)	
H17D	0.460129	0.098998	1.075327	0.06*	
C18D	0.45666 (6)	0.2178 (2)	1.02390 (14)	0.0416 (7)	
H18D	0.462119	0.186141	0.992184	0.05*	
C20D	0.43237 (5)	0.46110 (18)	0.84332 (10)	0.0238 (5)	
H20G	0.448677	0.431567	0.829094	0.029*	
H20H	0.419994	0.408437	0.852654	0.029*	
C21D	0.41357 (5)	0.52170 (18)	0.79378 (10)	0.0223 (5)	
H21D	0.397292	0.551977	0.808558	0.027*	
C22D	0.39952 (5)	0.45731 (19)	0.74098 (11)	0.0257 (5)	
H22G	0.414599	0.442938	0.718817	0.031*	
H22H	0.393608	0.395749	0.755884	0.031*	
C23D	0.37293 (5)	0.50103 (17)	0.69956 (10)	0.0220 (5)	
C24D	0.34540 (6)	0.49365 (19)	0.71243 (11)	0.0274 (5)	
H24D	0.343764	0.46114	0.747154	0.033*	
C25D	0.32033 (6)	0.5328 (2)	0.67554 (12)	0.0317 (6)	
H25D	0.301708	0.527513	0.685105	0.038*	
C26D	0.32252 (6)	0.57955 (19)	0.62487 (12)	0.0319 (6)	
H26D	0.305332	0.605637	0.599121	0.038*	
C27D	0.34955 (6)	0.58840 (19)	0.61163 (11)	0.0299 (6)	
H27D	0.351021	0.621161	0.576883	0.036*	
C28D	0.37480 (6)	0.54958 (18)	0.64888 (11)	0.0254 (5)	
H28D	0.393422	0.556392	0.639517	0.031*	
C29D	0.42202 (5)	0.68792 (18)	0.76352 (10)	0.0236 (5)	
C30D	0.44528 (6)	0.75431 (18)	0.74796 (11)	0.0258 (5)	
H30D	0.46475	0.720303	0.758617	0.031*	
C31D	0.44830 (7)	0.8468 (2)	0.78391 (13)	0.0373 (7)	
H31D	0.42881	0.88074	0.774769	0.045*	
C32D	0.45665 (8)	0.8211 (3)	0.84893 (14)	0.0493 (8)	
H32J	0.441769	0.777472	0.857678	0.074*	
H32K	0.457527	0.879632	0.872478	0.074*	
H32L	0.47603	0.789545	0.858559	0.074*	
C33D	0.47166 (8)	0.9130 (2)	0.76852 (17)	0.0527 (9)	
H33J	0.490746	0.879688	0.776476	0.079*	
H33K	0.47353	0.971342	0.792508	0.079*	
H33L	0.465614	0.930213	0.726745	0.079*	
C34D	0.44645 (6)	0.7006 (2)	0.65107 (11)	0.0300 (6)	
C35D	0.41441 (7)	0.8391 (2)	0.65765 (13)	0.0396 (7)	
H35G	0.423767	0.901529	0.653152	0.047*	
H35H	0.401148	0.848624	0.684398	0.047*	
C36D	0.39678 (8)	0.8068 (2)	0.59960 (16)	0.0540 (9)	
H36G	0.383669	0.859587	0.580526	0.065*	
H36H	0.384178	0.751945	0.60489	0.065*	
C37D	0.41648 (10)	0.7774 (3)	0.56110 (14)	0.0620 (11)	
H37G	0.404497	0.748645	0.524334	0.074*	
H37H	0.426774	0.83442	0.550528	0.074*	
N1D	0.40871 (4)	0.47069 (16)	0.95273 (9)	0.0255 (4)	
H1D	0.397479	0.422882	0.935751	0.031*	
N2D	0.43152 (4)	0.59773 (15)	0.77781 (9)	0.0229 (4)	
H2D	0.449896	0.583842	0.777457	0.028*	
N3D	0.43748 (5)	0.76918 (15)	0.68385 (9)	0.0258 (4)	
N4D	0.43803 (6)	0.7082 (2)	0.59170 (10)	0.0458 (6)	
H4D	0.446083	0.669007	0.570541	0.055*	
O1D	0.35289 (4)	0.44264 (12)	0.95483 (7)	0.0233 (4)	
O2D	0.40958 (4)	0.61521 (15)	0.99845 (10)	0.0429 (5)	
O3D	0.47712 (4)	0.52957 (16)	0.91070 (8)	0.0344 (4)	
H3D	0.481973	0.530093	0.878425	0.052*	
O4D	0.39688 (4)	0.71599 (14)	0.76210 (8)	0.0322 (4)	
O5D	0.46217 (5)	0.63243 (15)	0.67483 (9)	0.0436 (5)	
C1	0.49162 (8)	0.4207 (3)	0.72586 (18)	0.0544 (9)	
H1E	0.498245	0.355698	0.717751	0.065*	
H1F	0.469917	0.41826	0.720876	0.065*	
C2	0.50580 (7)	0.4462 (3)	0.78815 (17)	0.0513 (9)	
H2E	0.503169	0.392341	0.813963	0.062*	
H2F	0.527303	0.455088	0.792411	0.062*	
O1	0.49821 (5)	0.48731 (17)	0.68271 (12)	0.0509 (6)	
H1	0.492255	0.542466	0.688656	0.076*	
O2	0.49398 (4)	0.53076 (15)	0.80693 (9)	0.0358 (4)	
H2	0.504464	0.578002	0.802664	0.054*	
O3	0.54170 (18)	0.9506 (7)	0.9927 (5)	0.0462 (15)	0.5
H3O	0.545567	0.894564	0.983576	0.069*	0.5
C3	0.51078 (16)	0.9648 (6)	0.9772 (3)	0.0455 (13)	0.5
H31	0.50252	0.935039	0.938292	0.055*	0.5
H32	0.506699	1.034507	0.973371	0.055*	0.5
C3'	0.49543 (16)	0.9242 (6)	1.0202 (3)	0.0455 (13)	0.5
H31'	0.500024	0.854931	1.025952	0.055*	0.5
H32'	0.502298	0.956863	1.058651	0.055*	0.5
O3'	0.46396 (18)	0.9373 (7)	0.9985 (5)	0.0462 (15)	0.5
H3O'	0.455331	0.884413	0.998774	0.069*	0.5
O1W	0.75091 (4)	0.00874 (12)	0.71917 (9)	0.0314 (4)	
H1WA	0.758202	0.062916	0.73468	0.047*	
H1WB	0.732314	0.020917	0.703991	0.047*	
O2W	0.75284 (4)	0.51347 (13)	0.79013 (9)	0.0347 (4)	
H2WA	0.744248	0.565445	0.773766	0.052*	
H2WB	0.771607	0.527248	0.799036	0.052*	
O3W	0.54629 (4)	0.75136 (15)	0.96751 (10)	0.0404 (5)	
H3WA	0.561049	0.712262	0.979878	0.061*	
H3WB	0.531059	0.721069	0.973983	0.061*	
O4W	0.5	0.65511 (19)	1	0.0296 (6)	
H4WA	0.510526	0.617334	1.026584	0.044*	0.5
H4WB	0.495289	0.619954	0.968397	0.044*	0.5

### (2S)-N-[(2S,4S,5S)-5-[2-(2,6-Dimethylphenoxy)acetamido]-4-hydroxy-1,6-diphenylhexan-2-yl]-3-methyl-2-(2-oxo-1,3-diazinan-1-yl)butanamide–ethane-1,2-diol–water (8/2/1/7) Atomic displacement parameters (Å^2^)

**Table:**

	*U*^11^	*U*^22^	*U*^33^	*U*^12^	*U*^13^	*U*^23^
C1A	0.0271 (12)	0.0150 (11)	0.0138 (10)	−0.0041 (9)	0.0040 (9)	−0.0020 (8)
C2A	0.0276 (13)	0.0179 (12)	0.0231 (12)	0.0002 (9)	0.0061 (10)	−0.0042 (9)
C3A	0.0397 (16)	0.0289 (15)	0.0361 (15)	−0.0031 (11)	0.0182 (12)	−0.0056 (11)
C4A	0.0574 (19)	0.0309 (15)	0.0273 (14)	−0.0166 (13)	0.0234 (13)	−0.0081 (11)
C5A	0.061 (2)	0.0280 (15)	0.0127 (11)	−0.0172 (13)	0.0011 (11)	0.0007 (10)
C6A	0.0328 (14)	0.0211 (12)	0.0191 (12)	−0.0077 (10)	−0.0032 (10)	0.0009 (9)
C7A	0.0278 (13)	0.0329 (15)	0.0305 (14)	0.0070 (11)	0.0010 (11)	−0.0006 (11)
C8A	0.0352 (16)	0.056 (2)	0.0365 (16)	0.0002 (14)	−0.0115 (13)	0.0159 (14)
C9A	0.0285 (13)	0.0200 (12)	0.0173 (11)	−0.0096 (9)	0.0033 (9)	−0.0026 (9)
C10A	0.0170 (11)	0.0180 (11)	0.0170 (11)	−0.0012 (8)	0.0032 (8)	−0.0021 (8)
C11A	0.0195 (11)	0.0204 (12)	0.0136 (10)	0.0004 (9)	0.0031 (9)	0.0009 (9)
C12A	0.0203 (12)	0.0332 (14)	0.0190 (11)	0.0052 (10)	0.0028 (9)	−0.0038 (10)
C13A	0.0197 (12)	0.0242 (13)	0.0210 (11)	0.0043 (9)	0.0037 (9)	−0.0037 (9)
C14A	0.0272 (14)	0.0311 (15)	0.0485 (17)	0.0060 (11)	0.0123 (12)	0.0056 (13)
C15A	0.0234 (14)	0.0343 (17)	0.085 (3)	−0.0031 (12)	0.0138 (15)	−0.0059 (16)
C16A	0.0241 (15)	0.052 (2)	0.060 (2)	0.0054 (13)	−0.0100 (14)	−0.0196 (17)
C17A	0.0418 (17)	0.052 (2)	0.0308 (15)	0.0178 (15)	−0.0072 (13)	−0.0018 (13)
C18A	0.0263 (13)	0.0317 (14)	0.0280 (13)	0.0066 (10)	0.0034 (11)	0.0028 (11)
C19A	0.0186 (11)	0.0172 (11)	0.0141 (10)	−0.0006 (8)	0.0012 (8)	0.0009 (8)
C20A	0.0184 (11)	0.0182 (11)	0.0152 (10)	0.0013 (8)	0.0021 (8)	−0.0009 (8)
C21A	0.0177 (11)	0.0146 (11)	0.0175 (10)	0.0018 (8)	0.0037 (9)	0.0007 (8)
C22A	0.0185 (11)	0.0189 (12)	0.0187 (11)	0.0007 (9)	0.0033 (9)	0.0012 (9)
C23A	0.0178 (11)	0.0216 (12)	0.0156 (10)	0.0024 (9)	0.0035 (9)	0.0012 (9)
C24A	0.0229 (12)	0.0199 (12)	0.0203 (11)	0.0035 (9)	0.0009 (9)	0.0009 (9)
C25A	0.0278 (13)	0.0235 (13)	0.0209 (12)	−0.0037 (10)	0.0007 (10)	−0.0029 (10)
C26A	0.0235 (12)	0.0339 (14)	0.0178 (11)	0.0007 (10)	−0.0012 (9)	0.0004 (10)
C27A	0.0280 (13)	0.0255 (13)	0.0243 (12)	0.0068 (10)	−0.0013 (10)	0.0031 (10)
C28A	0.0282 (13)	0.0182 (12)	0.0238 (12)	0.0040 (9)	0.0013 (10)	−0.0001 (9)
C29A	0.0174 (11)	0.0155 (12)	0.0188 (11)	0.0021 (8)	0.0006 (9)	0.0027 (8)
C30A	0.0171 (11)	0.0150 (11)	0.0196 (11)	0.0004 (8)	0.0017 (9)	0.0021 (8)
C31A	0.0219 (12)	0.0204 (12)	0.0240 (12)	−0.0009 (9)	0.0067 (9)	0.0034 (9)
C32A	0.0347 (15)	0.0501 (18)	0.0215 (12)	−0.0090 (13)	0.0014 (11)	0.0069 (12)
C33A	0.0474 (17)	0.0301 (15)	0.0417 (16)	0.0049 (12)	0.0282 (14)	0.0050 (12)
C34A	0.0162 (11)	0.0150 (11)	0.0202 (11)	0.0025 (8)	0.0064 (9)	−0.0012 (9)
C35A	0.0223 (12)	0.0166 (12)	0.0346 (14)	−0.0026 (9)	0.0003 (10)	0.0005 (10)
C36A	0.0403 (16)	0.0229 (14)	0.0316 (14)	−0.0094 (11)	0.0024 (12)	−0.0046 (11)
C37A	0.0354 (15)	0.0241 (14)	0.0368 (15)	−0.0052 (11)	−0.0109 (12)	0.0004 (11)
N1A	0.0174 (9)	0.0163 (9)	0.0135 (9)	−0.0020 (7)	0.0017 (7)	0.0002 (7)
N2A	0.0167 (9)	0.0122 (9)	0.0199 (9)	−0.0003 (7)	0.0032 (7)	−0.0004 (7)
N3A	0.0160 (9)	0.0135 (9)	0.0229 (10)	0.0000 (7)	0.0004 (8)	0.0019 (7)
N4A	0.0260 (11)	0.0169 (10)	0.0252 (10)	0.0002 (8)	−0.0047 (8)	0.0016 (8)
O1A	0.0266 (9)	0.0159 (8)	0.0165 (8)	−0.0030 (6)	0.0062 (6)	−0.0006 (6)
O2A	0.0303 (9)	0.0242 (9)	0.0212 (8)	−0.0106 (7)	0.0083 (7)	−0.0034 (7)
O3A	0.0237 (8)	0.0179 (8)	0.0177 (8)	0.0004 (6)	0.0008 (7)	−0.0009 (6)
O4A	0.0221 (9)	0.0130 (9)	0.0424 (10)	0.0005 (6)	0.0100 (7)	0.0041 (7)
O5A	0.0198 (8)	0.0146 (8)	0.0205 (8)	−0.0014 (6)	0.0020 (6)	0.0009 (6)
C1B	0.0269 (12)	0.0136 (11)	0.0155 (11)	0.0041 (9)	0.0031 (9)	0.0004 (8)
C2B	0.0333 (14)	0.0156 (12)	0.0224 (12)	0.0078 (9)	−0.0044 (10)	−0.0015 (9)
C3B	0.0569 (18)	0.0221 (13)	0.0136 (11)	0.0149 (12)	−0.0009 (11)	−0.0004 (9)
C4B	0.0558 (19)	0.0267 (14)	0.0233 (13)	0.0126 (12)	0.0183 (12)	0.0069 (10)
C5B	0.0353 (14)	0.0252 (13)	0.0296 (13)	0.0028 (10)	0.0135 (11)	0.0062 (10)
C6B	0.0290 (13)	0.0150 (11)	0.0217 (12)	0.0017 (9)	0.0041 (10)	0.0033 (9)
C7B	0.0359 (16)	0.0345 (16)	0.0380 (16)	0.0043 (12)	−0.0118 (12)	−0.0120 (12)
C8B	0.0287 (14)	0.0295 (14)	0.0273 (13)	−0.0049 (10)	0.0010 (11)	−0.0013 (11)
C9B	0.0330 (14)	0.0241 (13)	0.0214 (12)	0.0133 (10)	0.0048 (10)	0.0040 (10)
C10B	0.0166 (11)	0.0179 (11)	0.0209 (11)	0.0009 (9)	0.0029 (9)	0.0028 (9)
C11B	0.0187 (11)	0.0198 (12)	0.0168 (11)	0.0004 (9)	0.0032 (9)	−0.0007 (9)
C12B	0.0228 (13)	0.0320 (14)	0.0218 (12)	−0.0056 (10)	0.0033 (10)	0.0049 (10)
C13B	0.0186 (12)	0.0252 (13)	0.0315 (13)	−0.0052 (9)	0.0040 (10)	0.0071 (10)
C14B	0.0312 (16)	0.0347 (17)	0.076 (2)	−0.0039 (12)	0.0217 (15)	0.0026 (15)
C15B	0.0267 (18)	0.049 (2)	0.149 (5)	0.0064 (16)	0.031 (2)	0.027 (3)
C16B	0.0234 (18)	0.072 (3)	0.114 (4)	−0.0115 (18)	−0.016 (2)	0.048 (3)
C17B	0.048 (2)	0.071 (3)	0.050 (2)	−0.033 (2)	−0.0190 (16)	0.0174 (18)
C18B	0.0326 (15)	0.0385 (16)	0.0344 (15)	−0.0131 (12)	0.0012 (12)	0.0038 (12)
C19B	0.0179 (11)	0.0192 (11)	0.0160 (10)	0.0000 (9)	0.0018 (9)	0.0000 (9)
C20B	0.0182 (11)	0.0193 (11)	0.0166 (10)	−0.0014 (8)	0.0020 (9)	0.0011 (9)
C21B	0.0164 (11)	0.0172 (11)	0.0183 (11)	−0.0016 (8)	0.0046 (9)	−0.0010 (8)
C22B	0.0176 (11)	0.0223 (12)	0.0190 (11)	−0.0006 (9)	0.0032 (9)	−0.0025 (9)
C23B	0.0188 (11)	0.0231 (12)	0.0154 (10)	−0.0017 (9)	0.0071 (9)	−0.0018 (9)
C24B	0.0269 (13)	0.0190 (12)	0.0211 (11)	−0.0039 (9)	0.0059 (10)	−0.0011 (9)
C25B	0.0264 (13)	0.0283 (14)	0.0230 (12)	−0.0083 (10)	0.0039 (10)	−0.0044 (10)
C26B	0.0216 (12)	0.0402 (15)	0.0193 (11)	−0.0020 (11)	0.0019 (9)	−0.0007 (11)
C27B	0.0284 (13)	0.0275 (14)	0.0232 (12)	0.0044 (10)	0.0065 (10)	0.0029 (10)
C28B	0.0232 (12)	0.0201 (12)	0.0225 (11)	−0.0037 (9)	0.0052 (9)	−0.0014 (9)
C29B	0.0213 (12)	0.0166 (12)	0.0170 (10)	−0.0033 (9)	0.0040 (9)	−0.0036 (8)
C30B	0.0176 (11)	0.0149 (11)	0.0203 (11)	−0.0016 (8)	0.0047 (9)	−0.0029 (9)
C31B	0.0248 (12)	0.0201 (12)	0.0229 (12)	0.0012 (9)	0.0091 (10)	−0.0036 (9)
C32B	0.0324 (15)	0.0533 (19)	0.0230 (13)	−0.0017 (13)	0.0052 (11)	−0.0129 (12)
C33B	0.0288 (13)	0.0281 (14)	0.0290 (13)	0.0008 (10)	0.0135 (11)	0.0002 (10)
C34B	0.0192 (11)	0.0164 (11)	0.0177 (11)	−0.0026 (8)	0.0078 (9)	0.0002 (8)
C35B	0.0303 (14)	0.0162 (12)	0.0326 (13)	0.0031 (10)	0.0009 (11)	0.0001 (10)
C36B	0.0308 (14)	0.0266 (14)	0.0307 (13)	0.0082 (11)	0.0022 (11)	−0.0003 (11)
C37B	0.0244 (13)	0.0279 (14)	0.0240 (12)	0.0026 (10)	−0.0001 (10)	0.0012 (10)
N1B	0.0168 (9)	0.0157 (9)	0.0163 (9)	0.0033 (7)	0.0025 (7)	0.0016 (7)
N2B	0.0190 (10)	0.0134 (9)	0.0210 (9)	−0.0022 (7)	0.0059 (8)	−0.0002 (7)
N3B	0.0189 (10)	0.0147 (10)	0.0205 (9)	−0.0018 (7)	0.0029 (8)	−0.0014 (7)
N4B	0.0257 (10)	0.0179 (10)	0.0206 (10)	0.0000 (8)	−0.0007 (8)	−0.0022 (8)
O1B	0.0247 (8)	0.0156 (8)	0.0183 (8)	0.0049 (6)	0.0045 (6)	0.0031 (6)
O2B	0.0276 (9)	0.0235 (9)	0.0243 (8)	0.0105 (7)	0.0069 (7)	0.0039 (7)
O3B	0.0239 (8)	0.0171 (8)	0.0197 (8)	0.0016 (6)	0.0010 (7)	0.0022 (6)
O4B	0.0265 (9)	0.0154 (9)	0.0447 (11)	−0.0027 (7)	0.0150 (8)	−0.0072 (7)
O5B	0.0252 (8)	0.0137 (8)	0.0202 (8)	0.0006 (6)	0.0031 (6)	−0.0003 (6)
C1C	0.0436 (15)	0.0145 (12)	0.0255 (12)	0.0005 (10)	0.0104 (11)	0.0044 (10)
C2C	0.0423 (15)	0.0166 (12)	0.0219 (12)	0.0021 (10)	0.0065 (11)	0.0054 (9)
C3C	0.0417 (16)	0.0296 (15)	0.0305 (14)	0.0027 (12)	0.0124 (12)	0.0064 (11)
C4C	0.0401 (16)	0.0347 (16)	0.0415 (17)	−0.0036 (12)	0.0033 (13)	0.0107 (13)
C5C	0.058 (2)	0.0214 (14)	0.0312 (15)	−0.0094 (12)	−0.0056 (13)	0.0057 (11)
C6C	0.0568 (18)	0.0142 (12)	0.0260 (13)	−0.0012 (11)	0.0076 (12)	0.0017 (10)
C7C	0.0436 (16)	0.0234 (13)	0.0244 (13)	0.0023 (11)	0.0076 (11)	0.0014 (10)
C8C	0.084 (3)	0.0277 (16)	0.0300 (15)	0.0043 (15)	0.0130 (15)	−0.0031 (12)
C9C	0.060 (2)	0.0253 (15)	0.0490 (18)	0.0152 (13)	0.0204 (15)	0.0164 (13)
C10C	0.058 (2)	0.0346 (17)	0.0364 (16)	0.0194 (14)	0.0085 (14)	0.0120 (13)
C11C	0.0326 (15)	0.0376 (16)	0.0283 (14)	0.0115 (12)	−0.0109 (11)	−0.0046 (11)
C12C	0.0360 (15)	0.0403 (17)	0.0297 (14)	0.0042 (12)	−0.0062 (12)	−0.0056 (12)
C13C	0.0312 (15)	0.0438 (18)	0.0349 (15)	0.0100 (12)	−0.0103 (12)	−0.0100 (13)
C14C	0.090 (3)	0.047 (2)	0.0303 (16)	0.0213 (19)	0.0050 (16)	−0.0022 (14)
C15C	0.101 (3)	0.069 (3)	0.0325 (18)	0.040 (2)	0.0063 (19)	−0.0027 (17)
C16C	0.050 (2)	0.074 (3)	0.043 (2)	0.0288 (18)	−0.0166 (16)	−0.0268 (19)
C17C	0.0321 (17)	0.057 (2)	0.074 (3)	−0.0008 (15)	−0.0042 (16)	−0.035 (2)
C18C	0.0305 (16)	0.052 (2)	0.059 (2)	−0.0047 (13)	0.0043 (14)	−0.0216 (16)
C19C	0.0262 (14)	0.0464 (18)	0.0415 (16)	0.0151 (12)	−0.0085 (12)	−0.0112 (13)
C19D	0.0213 (12)	0.0301 (14)	0.0251 (12)	−0.0065 (10)	0.0041 (10)	−0.0027 (10)
C20C	0.0219 (13)	0.0360 (15)	0.0354 (14)	0.0048 (11)	0.0074 (11)	−0.0084 (12)
C21C	0.0216 (12)	0.0331 (14)	0.0268 (13)	0.0036 (10)	0.0078 (10)	−0.0009 (10)
C22C	0.0316 (14)	0.0289 (14)	0.0275 (13)	0.0011 (11)	0.0077 (11)	−0.0008 (11)
C23C	0.0343 (14)	0.0254 (13)	0.0226 (12)	0.0030 (11)	0.0043 (10)	0.0063 (10)
C24C	0.0374 (15)	0.0319 (15)	0.0306 (14)	0.0002 (12)	0.0094 (12)	0.0079 (11)
C25C	0.0392 (16)	0.0372 (17)	0.0426 (16)	−0.0105 (13)	0.0018 (13)	0.0137 (14)
C26C	0.060 (2)	0.0301 (16)	0.0323 (15)	−0.0124 (14)	−0.0040 (14)	0.0080 (12)
C27C	0.065 (2)	0.0301 (15)	0.0245 (14)	0.0025 (14)	0.0052 (13)	0.0017 (11)
C28C	0.0444 (16)	0.0346 (16)	0.0257 (13)	0.0049 (12)	0.0079 (12)	0.0048 (11)
C29C	0.0244 (13)	0.0353 (15)	0.0294 (13)	−0.0046 (11)	0.0017 (11)	−0.0001 (11)
C30C	0.0256 (13)	0.0297 (14)	0.0361 (14)	−0.0043 (10)	0.0047 (11)	−0.0018 (11)
C31C	0.0390 (16)	0.0344 (16)	0.0443 (17)	−0.0015 (12)	0.0016 (13)	0.0069 (13)
C32C	0.075 (3)	0.064 (2)	0.0409 (19)	−0.001 (2)	−0.0110 (17)	0.0048 (17)
C33C	0.0430 (18)	0.0380 (19)	0.076 (2)	0.0028 (14)	0.0056 (17)	0.0151 (17)
C34C	0.0273 (14)	0.0257 (14)	0.0405 (15)	−0.0029 (11)	0.0075 (11)	0.0022 (11)
C35C	0.068 (2)	0.071 (3)	0.0478 (19)	−0.049 (2)	0.0182 (17)	−0.0125 (18)
C36C	0.091 (3)	0.056 (2)	0.061 (2)	−0.049 (2)	0.028 (2)	−0.0174 (19)
C37C	0.112 (4)	0.066 (3)	0.052 (2)	−0.058 (3)	0.029 (2)	−0.020 (2)
N1C	0.0384 (13)	0.0278 (12)	0.0263 (11)	0.0127 (10)	−0.0006 (9)	0.0048 (9)
N2C	0.0209 (10)	0.0291 (12)	0.0293 (11)	−0.0006 (8)	0.0092 (8)	0.0007 (9)
N3C	0.0334 (12)	0.0271 (12)	0.0382 (13)	−0.0106 (9)	0.0078 (10)	−0.0027 (10)
N4C	0.0610 (17)	0.0454 (16)	0.0371 (14)	−0.0251 (13)	0.0156 (12)	−0.0030 (12)
O1C	0.0416 (11)	0.0204 (9)	0.0336 (10)	0.0076 (8)	0.0154 (8)	0.0089 (7)
O2C	0.0776 (18)	0.0577 (17)	0.0763 (18)	0.0331 (14)	0.0149 (14)	0.0436 (15)
O3C	0.0218 (11)	0.112 (2)	0.0660 (16)	0.0255 (13)	−0.0117 (10)	−0.0383 (16)
O4C	0.0291 (11)	0.0429 (12)	0.0617 (14)	−0.0074 (9)	0.0141 (10)	0.0052 (10)
O5C	0.0566 (14)	0.0504 (14)	0.0516 (13)	−0.0316 (11)	0.0164 (11)	−0.0025 (11)
C1D	0.0241 (12)	0.0144 (11)	0.0243 (12)	−0.0032 (9)	0.0091 (9)	−0.0035 (9)
C2D	0.0310 (13)	0.0133 (11)	0.0253 (12)	−0.0026 (9)	0.0042 (10)	−0.0006 (9)
C3D	0.0303 (14)	0.0174 (12)	0.0382 (15)	0.0000 (10)	0.0011 (11)	−0.0024 (10)
C4D	0.0269 (14)	0.0307 (15)	0.0506 (17)	−0.0049 (11)	0.0134 (12)	−0.0090 (13)
C5D	0.0388 (15)	0.0306 (15)	0.0343 (14)	−0.0076 (11)	0.0214 (12)	−0.0027 (11)
C6D	0.0353 (14)	0.0177 (12)	0.0244 (12)	−0.0047 (10)	0.0102 (10)	−0.0032 (9)
C7D	0.0526 (18)	0.0272 (14)	0.0243 (13)	−0.0056 (12)	0.0056 (12)	0.0030 (11)
C8D	0.0474 (17)	0.0281 (14)	0.0222 (13)	−0.0030 (12)	0.0082 (12)	0.0007 (10)
C9D	0.0309 (14)	0.0234 (13)	0.0371 (14)	−0.0061 (10)	0.0129 (11)	−0.0096 (11)
C10D	0.0299 (14)	0.0286 (14)	0.0292 (13)	−0.0074 (11)	0.0081 (11)	−0.0062 (11)
C11D	0.0216 (12)	0.0354 (15)	0.0202 (12)	−0.0051 (10)	0.0029 (9)	−0.0043 (10)
C12D	0.0282 (14)	0.0415 (16)	0.0232 (13)	0.0024 (11)	0.0038 (10)	0.0018 (11)
C13D	0.0295 (14)	0.0437 (17)	0.0229 (13)	−0.0053 (12)	−0.0011 (10)	0.0029 (11)
C14D	0.098 (3)	0.053 (2)	0.0298 (16)	−0.014 (2)	0.0190 (17)	−0.0025 (15)
C15D	0.124 (4)	0.074 (3)	0.0300 (18)	−0.034 (3)	0.024 (2)	−0.0057 (18)
C16D	0.085 (3)	0.069 (3)	0.0352 (18)	−0.022 (2)	0.0076 (18)	0.0208 (18)
C17D	0.0418 (18)	0.053 (2)	0.051 (2)	−0.0061 (15)	0.0035 (15)	0.0212 (16)
C18D	0.0327 (15)	0.0515 (19)	0.0384 (16)	−0.0027 (13)	0.0045 (12)	0.0063 (14)
C20D	0.0246 (12)	0.0261 (13)	0.0198 (11)	−0.0059 (9)	0.0041 (9)	0.0005 (10)
C21D	0.0200 (11)	0.0249 (13)	0.0221 (12)	−0.0037 (9)	0.0056 (9)	−0.0034 (10)
C22D	0.0258 (13)	0.0255 (13)	0.0233 (12)	−0.0016 (10)	0.0011 (10)	−0.0023 (10)
C23D	0.0259 (12)	0.0206 (12)	0.0187 (11)	−0.0044 (9)	0.0036 (9)	−0.0048 (9)
C24D	0.0296 (13)	0.0274 (13)	0.0265 (12)	−0.0066 (10)	0.0092 (10)	−0.0058 (10)
C25D	0.0244 (13)	0.0312 (14)	0.0396 (15)	−0.0028 (10)	0.0079 (11)	−0.0132 (12)
C26D	0.0317 (14)	0.0255 (14)	0.0329 (14)	0.0062 (10)	−0.0034 (11)	−0.0092 (11)
C27D	0.0429 (16)	0.0222 (13)	0.0210 (12)	0.0012 (11)	0.0010 (11)	−0.0021 (10)
C28D	0.0293 (13)	0.0243 (13)	0.0230 (12)	−0.0048 (10)	0.0068 (10)	−0.0039 (10)
C29D	0.0266 (13)	0.0242 (13)	0.0192 (11)	0.0026 (10)	0.0039 (9)	−0.0034 (9)
C30D	0.0287 (13)	0.0221 (13)	0.0257 (12)	0.0014 (10)	0.0047 (10)	−0.0017 (10)
C31D	0.0453 (17)	0.0259 (15)	0.0402 (16)	−0.0044 (12)	0.0096 (13)	−0.0104 (12)
C32D	0.059 (2)	0.0453 (19)	0.0409 (17)	−0.0128 (16)	0.0056 (15)	−0.0217 (15)
C33D	0.064 (2)	0.0318 (17)	0.064 (2)	−0.0181 (15)	0.0183 (18)	−0.0156 (16)
C34D	0.0306 (14)	0.0319 (15)	0.0267 (13)	−0.0008 (11)	0.0057 (11)	−0.0017 (11)
C35D	0.0392 (16)	0.0382 (17)	0.0412 (16)	0.0118 (13)	0.0095 (13)	0.0130 (13)
C36D	0.054 (2)	0.0367 (18)	0.057 (2)	−0.0008 (15)	−0.0150 (17)	0.0113 (15)
C37D	0.093 (3)	0.051 (2)	0.0312 (17)	0.007 (2)	−0.0066 (17)	0.0042 (15)
N1D	0.0229 (11)	0.0290 (12)	0.0252 (10)	−0.0067 (8)	0.0072 (8)	−0.0058 (9)
N2D	0.0193 (10)	0.0240 (11)	0.0255 (10)	0.0008 (8)	0.0056 (8)	0.0004 (8)
N3D	0.0278 (11)	0.0222 (11)	0.0262 (10)	0.0023 (8)	0.0044 (8)	0.0018 (8)
N4D	0.0643 (18)	0.0458 (16)	0.0256 (12)	0.0036 (13)	0.0076 (12)	−0.0052 (11)
O1D	0.0235 (9)	0.0190 (8)	0.0284 (9)	−0.0056 (7)	0.0083 (7)	−0.0056 (7)
O2D	0.0360 (11)	0.0394 (12)	0.0556 (13)	−0.0159 (9)	0.0155 (10)	−0.0210 (10)
O3D	0.0234 (9)	0.0539 (12)	0.0259 (9)	−0.0142 (8)	0.0060 (7)	−0.0060 (9)
O4D	0.0290 (10)	0.0305 (10)	0.0376 (10)	0.0066 (8)	0.0093 (8)	−0.0011 (8)
O5D	0.0528 (13)	0.0396 (12)	0.0360 (11)	0.0201 (10)	0.0063 (9)	−0.0052 (9)
C1	0.0438 (18)	0.0413 (19)	0.085 (3)	−0.0065 (15)	0.0299 (18)	−0.0123 (18)
C2	0.0384 (17)	0.048 (2)	0.072 (2)	0.0058 (14)	0.0208 (16)	0.0151 (17)
O1	0.0356 (11)	0.0382 (12)	0.0849 (17)	0.0044 (9)	0.0265 (11)	−0.0134 (12)
O2	0.0294 (10)	0.0375 (11)	0.0441 (11)	−0.0025 (8)	0.0159 (9)	0.0075 (9)
O3	0.043 (3)	0.037 (2)	0.054 (3)	0.0016 (19)	0.002 (3)	0.001 (2)
C3	0.039 (3)	0.039 (5)	0.051 (2)	−0.0005 (17)	−0.0045 (19)	0.0048 (18)
C3'	0.039 (3)	0.039 (5)	0.051 (2)	−0.0005 (17)	−0.0045 (19)	0.0048 (18)
O3'	0.043 (3)	0.037 (2)	0.054 (3)	0.0016 (19)	0.002 (3)	0.001 (2)
O1W	0.0221 (9)	0.0178 (9)	0.0515 (12)	−0.0009 (7)	0.0035 (8)	−0.0034 (8)
O2W	0.0289 (10)	0.0188 (9)	0.0536 (12)	0.0015 (7)	0.0046 (9)	0.0047 (8)
O3W	0.0326 (11)	0.0354 (11)	0.0541 (13)	0.0066 (8)	0.0124 (10)	0.0124 (10)
O4W	0.0290 (14)	0.0295 (14)	0.0267 (13)	0	0.0000 (11)	0

### (2S)-N-[(2S,4S,5S)-5-[2-(2,6-Dimethylphenoxy)acetamido]-4-hydroxy-1,6-diphenylhexan-2-yl]-3-methyl-2-(2-oxo-1,3-diazinan-1-yl)butanamide–ethane-1,2-diol–water (8/2/1/7) Geometric parameters (Å, º)

**Table:**

C1A—C2A	1.390 (3)	C7C—H7CC	0.98
C1A—C6A	1.394 (3)	C8C—H8CA	0.98
C1A—O1A	1.400 (3)	C8C—H8CB	0.98
C2A—C3A	1.386 (4)	C8C—H8CC	0.98
C2A—C7A	1.503 (3)	C9C—O1C	1.423 (3)
C3A—C4A	1.381 (4)	C9C—C10C	1.498 (5)
C3A—H3AA	0.95	C9C—H9CA	0.99
C4A—C5A	1.378 (5)	C9C—H9CB	0.99
C4A—H4AA	0.95	C10C—O2C	1.233 (4)
C5A—C6A	1.390 (4)	C10C—N1C	1.330 (4)
C5A—H5A	0.95	C11C—N1C	1.465 (4)
C6A—C8A	1.506 (4)	C11C—C12C	1.523 (4)
C7A—H7AA	0.98	C11C—C19C	1.526 (4)
C7A—H7AB	0.98	C11C—H11C	1
C7A—H7AC	0.98	C12C—C13C	1.512 (4)
C8A—H8AA	0.98	C12C—H12E	0.99
C8A—H8AB	0.98	C12C—H12F	0.99
C8A—H8AC	0.98	C13C—C14C	1.362 (5)
C9A—O1A	1.429 (3)	C13C—C18C	1.394 (5)
C9A—C10A	1.510 (3)	C14C—C15C	1.402 (5)
C9A—H9AA	0.99	C14C—H14C	0.95
C9A—H9AB	0.99	C15C—C16C	1.378 (6)
C10A—O2A	1.235 (3)	C15C—H15C	0.95
C10A—N1A	1.337 (3)	C16C—C17C	1.348 (6)
C11A—N1A	1.464 (3)	C16C—H16C	0.95
C11A—C19A	1.536 (3)	C17C—C18C	1.395 (5)
C11A—C12A	1.540 (3)	C17C—H17C	0.95
C11A—H11A	1	C18C—H18C	0.95
C12A—C13A	1.508 (3)	C19C—O3C	1.432 (4)
C12A—H12A	0.99	C19C—C20C	1.534 (4)
C12A—H12B	0.99	C19C—H19C	1
C13A—C18A	1.381 (4)	C19D—O3D	1.433 (3)
C13A—C14A	1.381 (4)	C19D—C11D	1.535 (4)
C14A—C15A	1.394 (4)	C19D—C20D	1.541 (3)
C14A—H14A	0.95	C19D—H19D	1
C15A—C16A	1.368 (5)	C20C—C21C	1.523 (4)
C15A—H15A	0.95	C20C—H20E	0.99
C16A—C17A	1.376 (5)	C20C—H20F	0.99
C16A—H16A	0.95	C21C—N2C	1.456 (3)
C17A—C18A	1.393 (4)	C21C—C22C	1.540 (4)
C17A—H17A	0.95	C21C—H21C	1
C18A—H18A	0.95	C22C—C23C	1.503 (4)
C19A—O3A	1.430 (3)	C22C—H22E	0.99
C19A—C20A	1.531 (3)	C22C—H22F	0.99
C19A—H19A	1	C23C—C28C	1.390 (4)
C20A—C21A	1.532 (3)	C23C—C24C	1.394 (4)
C20A—H20A	0.99	C24C—C25C	1.391 (4)
C20A—H20B	0.99	C24C—H24C	0.95
C21A—N2A	1.459 (3)	C25C—C26C	1.374 (5)
C21A—C22A	1.535 (3)	C25C—H25C	0.95
C21A—H21A	1	C26C—C27C	1.380 (5)
C22A—C23A	1.515 (3)	C26C—H26C	0.95
C22A—H22A	0.99	C27C—C28C	1.385 (4)
C22A—H22B	0.99	C27C—H27C	0.95
C23A—C24A	1.390 (3)	C28C—H28C	0.95
C23A—C28A	1.392 (3)	C29C—O4C	1.227 (3)
C24A—C25A	1.390 (3)	C29C—N2C	1.346 (4)
C24A—H24A	0.95	C29C—C30C	1.531 (4)
C25A—C26A	1.385 (4)	C30C—N3C	1.466 (3)
C25A—H25A	0.95	C30C—C31C	1.536 (4)
C26A—C27A	1.384 (4)	C30C—H30C	1
C26A—H26A	0.95	C31C—C33C	1.520 (5)
C27A—C28A	1.390 (3)	C31C—C32C	1.529 (5)
C27A—H27A	0.95	C31C—H31C	1
C28A—H28A	0.95	C32C—H32G	0.98
C29A—O4A	1.236 (3)	C32C—H32H	0.98
C29A—N2A	1.341 (3)	C32C—H32I	0.98
C29A—C30A	1.527 (3)	C33C—H33G	0.98
C30A—N3A	1.470 (3)	C33C—H33H	0.98
C30A—C31A	1.538 (3)	C33C—H33I	0.98
C30A—H30A	1	C34C—O5C	1.232 (3)
C31A—C33A	1.516 (4)	C34C—N4C	1.337 (4)
C31A—C32A	1.527 (3)	C34C—N3C	1.359 (4)
C31A—H31A	1	C35C—C36C	1.451 (5)
C32A—H32A	0.98	C35C—N3C	1.461 (4)
C32A—H32B	0.98	C35C—H35E	0.99
C32A—H32C	0.98	C35C—H35F	0.99
C33A—H33A	0.98	C36C—C37C	1.432 (5)
C33A—H33B	0.98	C36C—H36E	0.99
C33A—H33C	0.98	C36C—H36F	0.99
C34A—O5A	1.260 (3)	C37C—N4C	1.457 (4)
C34A—N4A	1.350 (3)	C37C—H37E	0.99
C34A—N3A	1.350 (3)	C37C—H37F	0.99
C35A—N3A	1.476 (3)	N1C—H1C	0.88
C35A—C36A	1.493 (4)	N2C—H2C	0.88
C35A—H35A	0.99	N4C—H4C	0.88
C35A—H35B	0.99	O3C—H3C	0.8134
C36A—C37A	1.513 (4)	C1D—C6D	1.392 (3)
C36A—H36A	0.99	C1D—C2D	1.394 (3)
C36A—H36B	0.99	C1D—O1D	1.396 (3)
C37A—N4A	1.453 (3)	C2D—C3D	1.390 (4)
C37A—H37A	0.99	C2D—C7D	1.507 (4)
C37A—H37B	0.99	C3D—C4D	1.386 (4)
N1A—H1A	0.88	C3D—H3DD	0.95
N2A—H2A	0.88	C4D—C5D	1.384 (4)
N4A—H4A	0.88	C4D—H4DD	0.95
O3A—H3A	0.84	C5D—C6D	1.390 (4)
C1B—C2B	1.395 (3)	C5D—H5D	0.95
C1B—C6B	1.397 (3)	C6D—C8D	1.512 (4)
C1B—O1B	1.401 (3)	C7D—H7DA	0.98
C2B—C3B	1.393 (4)	C7D—H7DB	0.98
C2B—C7B	1.504 (4)	C7D—H7DC	0.98
C3B—C4B	1.379 (4)	C8D—H8DA	0.98
C3B—H3BB	0.95	C8D—H8DB	0.98
C4B—C5B	1.388 (4)	C8D—H8DC	0.98
C4B—H4BB	0.95	C9D—O1D	1.426 (3)
C5B—C6B	1.388 (4)	C9D—C10D	1.499 (4)
C5B—H5B	0.95	C9D—H9DA	0.99
C6B—C8B	1.500 (3)	C9D—H9DB	0.99
C7B—H7BA	0.98	C10D—O2D	1.229 (3)
C7B—H7BB	0.98	C10D—N1D	1.336 (3)
C7B—H7BC	0.98	C11D—N1D	1.462 (3)
C8B—H8BA	0.98	C11D—C12D	1.523 (4)
C8B—H8BB	0.98	C11D—H11D	1
C8B—H8BC	0.98	C12D—C13D	1.512 (4)
C9B—O1B	1.425 (3)	C12D—H12G	0.99
C9B—C10B	1.510 (3)	C12D—H12H	0.99
C9B—H9BA	0.99	C13D—C14D	1.377 (5)
C9B—H9BB	0.99	C13D—C18D	1.384 (5)
C10B—O2B	1.230 (3)	C14D—C15D	1.401 (5)
C10B—N1B	1.342 (3)	C14D—H14D	0.95
C11B—N1B	1.463 (3)	C15D—C16D	1.374 (7)
C11B—C19B	1.538 (3)	C15D—H15D	0.95
C11B—C12B	1.538 (3)	C16D—C17D	1.346 (6)
C11B—H11B	1	C16D—H16D	0.95
C12B—C13B	1.501 (3)	C17D—C18D	1.384 (4)
C12B—H12C	0.99	C17D—H17D	0.95
C12B—H12D	0.99	C18D—H18D	0.95
C13B—C18B	1.383 (4)	C20D—C21D	1.527 (3)
C13B—C14B	1.386 (4)	C20D—H20G	0.99
C14B—C15B	1.386 (5)	C20D—H20H	0.99
C14B—H14B	0.95	C21D—N2D	1.454 (3)
C15B—C16B	1.375 (7)	C21D—C22D	1.539 (3)
C15B—H15B	0.95	C21D—H21D	1
C16B—C17B	1.363 (7)	C22D—C23D	1.504 (3)
C16B—H16B	0.95	C22D—H22G	0.99
C17B—C18B	1.392 (4)	C22D—H22H	0.99
C17B—H17B	0.95	C23D—C28D	1.387 (3)
C18B—H18B	0.95	C23D—C24D	1.391 (4)
C19B—O3B	1.427 (3)	C24D—C25D	1.385 (4)
C19B—C20B	1.528 (3)	C24D—H24D	0.95
C19B—H19B	1	C25D—C26D	1.379 (4)
C20B—C21B	1.536 (3)	C25D—H25D	0.95
C20B—H20C	0.99	C26D—C27D	1.374 (4)
C20B—H20D	0.99	C26D—H26D	0.95
C21B—N2B	1.462 (3)	C27D—C28D	1.393 (4)
C21B—C22B	1.537 (3)	C27D—H27D	0.95
C21B—H21B	1	C28D—H28D	0.95
C22B—C23B	1.514 (3)	C29D—O4D	1.228 (3)
C22B—H22C	0.99	C29D—N2D	1.347 (3)
C22B—H22D	0.99	C29D—C30D	1.534 (4)
C23B—C24B	1.391 (3)	C30D—N3D	1.471 (3)
C23B—C28B	1.394 (3)	C30D—C31D	1.526 (4)
C24B—C25B	1.390 (4)	C30D—H30D	1
C24B—H24B	0.95	C31D—C32D	1.520 (4)
C25B—C26B	1.386 (4)	C31D—C33D	1.535 (4)
C25B—H25B	0.95	C31D—H31D	1
C26B—C27B	1.389 (4)	C32D—H32J	0.98
C26B—H26B	0.95	C32D—H32K	0.98
C27B—C28B	1.387 (3)	C32D—H32L	0.98
C27B—H27B	0.95	C33D—H33J	0.98
C28B—H28B	0.95	C33D—H33K	0.98
C29B—O4B	1.236 (3)	C33D—H33L	0.98
C29B—N2B	1.335 (3)	C34D—O5D	1.245 (3)
C29B—C30B	1.533 (3)	C34D—N3D	1.354 (3)
C30B—N3B	1.471 (3)	C34D—N4D	1.354 (4)
C30B—C31B	1.536 (3)	C35D—N3D	1.470 (3)
C30B—H30B	1	C35D—C36D	1.477 (5)
C31B—C33B	1.516 (3)	C35D—H35G	0.99
C31B—C32B	1.525 (3)	C35D—H35H	0.99
C31B—H31B	1	C36D—C37D	1.492 (6)
C32B—H32D	0.98	C36D—H36G	0.99
C32B—H32E	0.98	C36D—H36H	0.99
C32B—H32F	0.98	C37D—N4D	1.450 (4)
C33B—H33D	0.98	C37D—H37G	0.99
C33B—H33E	0.98	C37D—H37H	0.99
C33B—H33F	0.98	N1D—H1D	0.88
C34B—O5B	1.262 (3)	N2D—H2D	0.88
C34B—N3B	1.349 (3)	N4D—H4D	0.88
C34B—N4B	1.352 (3)	O3D—H3D	0.84
C35B—N3B	1.471 (3)	C1—O1	1.459 (5)
C35B—C36B	1.510 (4)	C1—C2	1.491 (6)
C35B—H35C	0.99	C1—H1E	0.99
C35B—H35D	0.99	C1—H1F	0.99
C36B—C37B	1.499 (4)	C2—O2	1.415 (4)
C36B—H36C	0.99	C2—H2E	0.99
C36B—H36D	0.99	C2—H2F	0.99
C37B—N4B	1.455 (3)	O1—H1	0.84
C37B—H37C	0.99	O2—H2	0.84
C37B—H37D	0.99	O3—C3	1.411 (8)
N1B—H1B	0.88	O3—H3O	0.84
N2B—H2B	0.88	C3—C3'	1.482 (9)
N4B—H4B	0.88	C3—H31	0.99
O3B—H3B	0.84	C3—H32	0.99
C1C—O1C	1.393 (3)	C3'—O3'	1.442 (8)
C1C—C2C	1.397 (4)	C3'—H31'	0.99
C1C—C6C	1.398 (4)	C3'—H32'	0.99
C2C—C3C	1.393 (4)	O3'—H3O'	0.84
C2C—C7C	1.507 (4)	O1W—H1WA	0.8701
C3C—C4C	1.378 (4)	O1W—H1WB	0.8698
C3C—H3CC	0.95	O2W—H2WA	0.87
C4C—C5C	1.379 (5)	O2W—H2WB	0.8698
C4C—H4CC	0.95	O3W—H3WA	0.87
C5C—C6C	1.392 (4)	O3W—H3WB	0.87
C5C—H5C	0.95	O4W—H4WA	0.8699
C6C—C8C	1.502 (4)	O4W—H4WB	0.8701
C7C—H7CA	0.98	O4W—H4WA^i^	0.87 (12)
C7C—H7CB	0.98	O4W—H4WB^i^	0.87 (11)
			
C2A—C1A—C6A	122.8 (2)	C2C—C7C—H7CB	109.5
C2A—C1A—O1A	118.6 (2)	H7CA—C7C—H7CB	109.5
C6A—C1A—O1A	118.5 (2)	C2C—C7C—H7CC	109.5
C3A—C2A—C1A	117.6 (2)	H7CA—C7C—H7CC	109.5
C3A—C2A—C7A	121.4 (2)	H7CB—C7C—H7CC	109.5
C1A—C2A—C7A	121.0 (2)	C6C—C8C—H8CA	109.5
C4A—C3A—C2A	121.1 (3)	C6C—C8C—H8CB	109.5
C4A—C3A—H3AA	119.4	H8CA—C8C—H8CB	109.5
C2A—C3A—H3AA	119.4	C6C—C8C—H8CC	109.5
C5A—C4A—C3A	119.9 (2)	H8CA—C8C—H8CC	109.5
C5A—C4A—H4AA	120	H8CB—C8C—H8CC	109.5
C3A—C4A—H4AA	120	O1C—C9C—C10C	111.6 (2)
C4A—C5A—C6A	121.2 (2)	O1C—C9C—H9CA	109.3
C4A—C5A—H5A	119.4	C10C—C9C—H9CA	109.3
C6A—C5A—H5A	119.4	O1C—C9C—H9CB	109.3
C5A—C6A—C1A	117.3 (2)	C10C—C9C—H9CB	109.3
C5A—C6A—C8A	121.7 (2)	H9CA—C9C—H9CB	108
C1A—C6A—C8A	121.0 (2)	O2C—C10C—N1C	123.9 (3)
C2A—C7A—H7AA	109.5	O2C—C10C—C9C	118.8 (3)
C2A—C7A—H7AB	109.5	N1C—C10C—C9C	117.3 (2)
H7AA—C7A—H7AB	109.5	N1C—C11C—C12C	110.9 (2)
C2A—C7A—H7AC	109.5	N1C—C11C—C19C	108.9 (2)
H7AA—C7A—H7AC	109.5	C12C—C11C—C19C	113.8 (3)
H7AB—C7A—H7AC	109.5	N1C—C11C—H11C	107.7
C6A—C8A—H8AA	109.5	C12C—C11C—H11C	107.7
C6A—C8A—H8AB	109.5	C19C—C11C—H11C	107.7
H8AA—C8A—H8AB	109.5	C13C—C12C—C11C	116.4 (3)
C6A—C8A—H8AC	109.5	C13C—C12C—H12E	108.2
H8AA—C8A—H8AC	109.5	C11C—C12C—H12E	108.2
H8AB—C8A—H8AC	109.5	C13C—C12C—H12F	108.2
O1A—C9A—C10A	111.72 (18)	C11C—C12C—H12F	108.2
O1A—C9A—H9AA	109.3	H12E—C12C—H12F	107.3
C10A—C9A—H9AA	109.3	C14C—C13C—C18C	117.4 (3)
O1A—C9A—H9AB	109.3	C14C—C13C—C12C	123.6 (3)
C10A—C9A—H9AB	109.3	C18C—C13C—C12C	118.9 (3)
H9AA—C9A—H9AB	107.9	C13C—C14C—C15C	121.1 (4)
O2A—C10A—N1A	124.7 (2)	C13C—C14C—H14C	119.4
O2A—C10A—C9A	117.97 (19)	C15C—C14C—H14C	119.4
N1A—C10A—C9A	117.35 (19)	C16C—C15C—C14C	120.4 (4)
N1A—C11A—C19A	112.65 (18)	C16C—C15C—H15C	119.8
N1A—C11A—C12A	110.43 (18)	C14C—C15C—H15C	119.8
C19A—C11A—C12A	110.78 (18)	C17C—C16C—C15C	119.3 (3)
N1A—C11A—H11A	107.6	C17C—C16C—H16C	120.3
C19A—C11A—H11A	107.6	C15C—C16C—H16C	120.3
C12A—C11A—H11A	107.6	C16C—C17C—C18C	120.4 (4)
C13A—C12A—C11A	113.71 (19)	C16C—C17C—H17C	119.8
C13A—C12A—H12A	108.8	C18C—C17C—H17C	119.8
C11A—C12A—H12A	108.8	C13C—C18C—C17C	121.3 (4)
C13A—C12A—H12B	108.8	C13C—C18C—H18C	119.3
C11A—C12A—H12B	108.8	C17C—C18C—H18C	119.3
H12A—C12A—H12B	107.7	O3C—C19C—C11C	106.3 (2)
C18A—C13A—C14A	118.6 (2)	O3C—C19C—C20C	111.4 (3)
C18A—C13A—C12A	120.6 (2)	C11C—C19C—C20C	113.9 (2)
C14A—C13A—C12A	120.8 (2)	O3C—C19C—H19C	108.4
C13A—C14A—C15A	120.6 (3)	C11C—C19C—H19C	108.4
C13A—C14A—H14A	119.7	C20C—C19C—H19C	108.4
C15A—C14A—H14A	119.7	O3D—C19D—C11D	106.10 (19)
C16A—C15A—C14A	120.2 (3)	O3D—C19D—C20D	113.0 (2)
C16A—C15A—H15A	119.9	C11D—C19D—C20D	113.0 (2)
C14A—C15A—H15A	119.9	O3D—C19D—H19D	108.2
C15A—C16A—C17A	119.9 (3)	C11D—C19D—H19D	108.2
C15A—C16A—H16A	120	C20D—C19D—H19D	108.2
C17A—C16A—H16A	120	C21C—C20C—C19C	114.2 (2)
C16A—C17A—C18A	119.9 (3)	C21C—C20C—H20E	108.7
C16A—C17A—H17A	120.1	C19C—C20C—H20E	108.7
C18A—C17A—H17A	120.1	C21C—C20C—H20F	108.7
C13A—C18A—C17A	120.8 (3)	C19C—C20C—H20F	108.7
C13A—C18A—H18A	119.6	H20E—C20C—H20F	107.6
C17A—C18A—H18A	119.6	N2C—C21C—C20C	109.8 (2)
O3A—C19A—C20A	110.69 (18)	N2C—C21C—C22C	111.3 (2)
O3A—C19A—C11A	113.17 (18)	C20C—C21C—C22C	109.0 (2)
C20A—C19A—C11A	112.91 (18)	N2C—C21C—H21C	108.9
O3A—C19A—H19A	106.5	C20C—C21C—H21C	108.9
C20A—C19A—H19A	106.5	C22C—C21C—H21C	108.9
C11A—C19A—H19A	106.5	C23C—C22C—C21C	115.0 (2)
C19A—C20A—C21A	113.18 (18)	C23C—C22C—H22E	108.5
C19A—C20A—H20A	108.9	C21C—C22C—H22E	108.5
C21A—C20A—H20A	108.9	C23C—C22C—H22F	108.5
C19A—C20A—H20B	108.9	C21C—C22C—H22F	108.5
C21A—C20A—H20B	108.9	H22E—C22C—H22F	107.5
H20A—C20A—H20B	107.8	C28C—C23C—C24C	118.3 (3)
N2A—C21A—C20A	110.24 (17)	C28C—C23C—C22C	121.4 (2)
N2A—C21A—C22A	108.84 (17)	C24C—C23C—C22C	120.3 (2)
C20A—C21A—C22A	112.51 (18)	C25C—C24C—C23C	120.6 (3)
N2A—C21A—H21A	108.4	C25C—C24C—H24C	119.7
C20A—C21A—H21A	108.4	C23C—C24C—H24C	119.7
C22A—C21A—H21A	108.4	C26C—C25C—C24C	120.1 (3)
C23A—C22A—C21A	114.05 (19)	C26C—C25C—H25C	119.9
C23A—C22A—H22A	108.7	C24C—C25C—H25C	119.9
C21A—C22A—H22A	108.7	C25C—C26C—C27C	120.0 (3)
C23A—C22A—H22B	108.7	C25C—C26C—H26C	120
C21A—C22A—H22B	108.7	C27C—C26C—H26C	120
H22A—C22A—H22B	107.6	C26C—C27C—C28C	120.1 (3)
C24A—C23A—C28A	118.2 (2)	C26C—C27C—H27C	119.9
C24A—C23A—C22A	120.7 (2)	C28C—C27C—H27C	119.9
C28A—C23A—C22A	121.0 (2)	C27C—C28C—C23C	120.9 (3)
C23A—C24A—C25A	121.5 (2)	C27C—C28C—H28C	119.6
C23A—C24A—H24A	119.3	C23C—C28C—H28C	119.6
C25A—C24A—H24A	119.3	O4C—C29C—N2C	123.5 (3)
C26A—C25A—C24A	119.4 (2)	O4C—C29C—C30C	121.9 (3)
C26A—C25A—H25A	120.3	N2C—C29C—C30C	114.6 (2)
C24A—C25A—H25A	120.3	N3C—C30C—C29C	109.4 (2)
C27A—C26A—C25A	120.0 (2)	N3C—C30C—C31C	113.4 (2)
C27A—C26A—H26A	120	C29C—C30C—C31C	111.1 (2)
C25A—C26A—H26A	120	N3C—C30C—H30C	107.6
C26A—C27A—C28A	120.1 (2)	C29C—C30C—H30C	107.6
C26A—C27A—H27A	119.9	C31C—C30C—H30C	107.6
C28A—C27A—H27A	119.9	C33C—C31C—C32C	110.7 (3)
C27A—C28A—C23A	120.7 (2)	C33C—C31C—C30C	110.5 (3)
C27A—C28A—H28A	119.6	C32C—C31C—C30C	109.7 (3)
C23A—C28A—H28A	119.6	C33C—C31C—H31C	108.6
O4A—C29A—N2A	123.7 (2)	C32C—C31C—H31C	108.6
O4A—C29A—C30A	121.0 (2)	C30C—C31C—H31C	108.6
N2A—C29A—C30A	115.25 (19)	C31C—C32C—H32G	109.5
N3A—C30A—C29A	107.96 (18)	C31C—C32C—H32H	109.5
N3A—C30A—C31A	111.63 (18)	H32G—C32C—H32H	109.5
C29A—C30A—C31A	112.72 (18)	C31C—C32C—H32I	109.5
N3A—C30A—H30A	108.1	H32G—C32C—H32I	109.5
C29A—C30A—H30A	108.1	H32H—C32C—H32I	109.5
C31A—C30A—H30A	108.1	C31C—C33C—H33G	109.5
C33A—C31A—C32A	111.8 (2)	C31C—C33C—H33H	109.5
C33A—C31A—C30A	110.3 (2)	H33G—C33C—H33H	109.5
C32A—C31A—C30A	109.70 (19)	C31C—C33C—H33I	109.5
C33A—C31A—H31A	108.3	H33G—C33C—H33I	109.5
C32A—C31A—H31A	108.3	H33H—C33C—H33I	109.5
C30A—C31A—H31A	108.3	O5C—C34C—N4C	120.3 (3)
C31A—C32A—H32A	109.5	O5C—C34C—N3C	120.7 (3)
C31A—C32A—H32B	109.5	N4C—C34C—N3C	119.0 (2)
H32A—C32A—H32B	109.5	C36C—C35C—N3C	112.2 (3)
C31A—C32A—H32C	109.5	C36C—C35C—H35E	109.2
H32A—C32A—H32C	109.5	N3C—C35C—H35E	109.2
H32B—C32A—H32C	109.5	C36C—C35C—H35F	109.2
C31A—C33A—H33A	109.5	N3C—C35C—H35F	109.2
C31A—C33A—H33B	109.5	H35E—C35C—H35F	107.9
H33A—C33A—H33B	109.5	C37C—C36C—C35C	116.6 (3)
C31A—C33A—H33C	109.5	C37C—C36C—H36E	108.1
H33A—C33A—H33C	109.5	C35C—C36C—H36E	108.1
H33B—C33A—H33C	109.5	C37C—C36C—H36F	108.1
O5A—C34A—N4A	118.9 (2)	C35C—C36C—H36F	108.1
O5A—C34A—N3A	123.0 (2)	H36E—C36C—H36F	107.3
N4A—C34A—N3A	118.1 (2)	C36C—C37C—N4C	111.7 (3)
N3A—C35A—C36A	110.8 (2)	C36C—C37C—H37E	109.3
N3A—C35A—H35A	109.5	N4C—C37C—H37E	109.3
C36A—C35A—H35A	109.5	C36C—C37C—H37F	109.3
N3A—C35A—H35B	109.5	N4C—C37C—H37F	109.3
C36A—C35A—H35B	109.5	H37E—C37C—H37F	107.9
H35A—C35A—H35B	108.1	C10C—N1C—C11C	121.4 (2)
C35A—C36A—C37A	110.4 (2)	C10C—N1C—H1C	119.3
C35A—C36A—H36A	109.6	C11C—N1C—H1C	119.3
C37A—C36A—H36A	109.6	C29C—N2C—C21C	124.7 (2)
C35A—C36A—H36B	109.6	C29C—N2C—H2C	117.6
C37A—C36A—H36B	109.6	C21C—N2C—H2C	117.6
H36A—C36A—H36B	108.1	C34C—N3C—C35C	119.9 (2)
N4A—C37A—C36A	110.0 (2)	C34C—N3C—C30C	118.5 (2)
N4A—C37A—H37A	109.7	C35C—N3C—C30C	119.7 (2)
C36A—C37A—H37A	109.7	C34C—N4C—C37C	125.2 (3)
N4A—C37A—H37B	109.7	C34C—N4C—H4C	117.4
C36A—C37A—H37B	109.7	C37C—N4C—H4C	117.4
H37A—C37A—H37B	108.2	C1C—O1C—C9C	114.3 (2)
C10A—N1A—C11A	122.12 (19)	C19C—O3C—H3C	105.5
C10A—N1A—H1A	118.9	C6D—C1D—C2D	123.2 (2)
C11A—N1A—H1A	118.9	C6D—C1D—O1D	118.4 (2)
C29A—N2A—C21A	123.53 (19)	C2D—C1D—O1D	118.3 (2)
C29A—N2A—H2A	118.2	C3D—C2D—C1D	117.4 (2)
C21A—N2A—H2A	118.2	C3D—C2D—C7D	121.7 (2)
C34A—N3A—C30A	120.59 (18)	C1D—C2D—C7D	121.0 (2)
C34A—N3A—C35A	121.77 (19)	C4D—C3D—C2D	120.8 (2)
C30A—N3A—C35A	117.61 (18)	C4D—C3D—H3DD	119.6
C34A—N4A—C37A	125.9 (2)	C2D—C3D—H3DD	119.6
C34A—N4A—H4A	117	C5D—C4D—C3D	120.2 (3)
C37A—N4A—H4A	117	C5D—C4D—H4DD	119.9
C1A—O1A—C9A	114.55 (17)	C3D—C4D—H4DD	119.9
C19A—O3A—H3A	109.5	C4D—C5D—C6D	121.0 (3)
C2B—C1B—C6B	122.8 (2)	C4D—C5D—H5D	119.5
C2B—C1B—O1B	119.3 (2)	C6D—C5D—H5D	119.5
C6B—C1B—O1B	117.87 (19)	C5D—C6D—C1D	117.3 (2)
C3B—C2B—C1B	117.4 (2)	C5D—C6D—C8D	120.9 (2)
C3B—C2B—C7B	121.7 (2)	C1D—C6D—C8D	121.8 (2)
C1B—C2B—C7B	120.9 (2)	C2D—C7D—H7DA	109.5
C4B—C3B—C2B	121.2 (2)	C2D—C7D—H7DB	109.5
C4B—C3B—H3BB	119.4	H7DA—C7D—H7DB	109.5
C2B—C3B—H3BB	119.4	C2D—C7D—H7DC	109.5
C3B—C4B—C5B	120.0 (2)	H7DA—C7D—H7DC	109.5
C3B—C4B—H4BB	120	H7DB—C7D—H7DC	109.5
C5B—C4B—H4BB	120	C6D—C8D—H8DA	109.5
C6B—C5B—C4B	121.1 (3)	C6D—C8D—H8DB	109.5
C6B—C5B—H5B	119.4	H8DA—C8D—H8DB	109.5
C4B—C5B—H5B	119.4	C6D—C8D—H8DC	109.5
C5B—C6B—C1B	117.5 (2)	H8DA—C8D—H8DC	109.5
C5B—C6B—C8B	121.5 (2)	H8DB—C8D—H8DC	109.5
C1B—C6B—C8B	121.0 (2)	O1D—C9D—C10D	111.3 (2)
C2B—C7B—H7BA	109.5	O1D—C9D—H9DA	109.4
C2B—C7B—H7BB	109.5	C10D—C9D—H9DA	109.4
H7BA—C7B—H7BB	109.5	O1D—C9D—H9DB	109.4
C2B—C7B—H7BC	109.5	C10D—C9D—H9DB	109.4
H7BA—C7B—H7BC	109.5	H9DA—C9D—H9DB	108
H7BB—C7B—H7BC	109.5	O2D—C10D—N1D	125.0 (2)
C6B—C8B—H8BA	109.5	O2D—C10D—C9D	117.7 (2)
C6B—C8B—H8BB	109.5	N1D—C10D—C9D	117.2 (2)
H8BA—C8B—H8BB	109.5	N1D—C11D—C12D	111.2 (2)
C6B—C8B—H8BC	109.5	N1D—C11D—C19D	108.8 (2)
H8BA—C8B—H8BC	109.5	C12D—C11D—C19D	112.8 (2)
H8BB—C8B—H8BC	109.5	N1D—C11D—H11D	107.9
O1B—C9B—C10B	112.45 (19)	C12D—C11D—H11D	107.9
O1B—C9B—H9BA	109.1	C19D—C11D—H11D	107.9
C10B—C9B—H9BA	109.1	C13D—C12D—C11D	116.8 (2)
O1B—C9B—H9BB	109.1	C13D—C12D—H12G	108.1
C10B—C9B—H9BB	109.1	C11D—C12D—H12G	108.1
H9BA—C9B—H9BB	107.8	C13D—C12D—H12H	108.1
O2B—C10B—N1B	124.6 (2)	C11D—C12D—H12H	108.1
O2B—C10B—C9B	118.1 (2)	H12G—C12D—H12H	107.3
N1B—C10B—C9B	117.3 (2)	C14D—C13D—C18D	117.3 (3)
N1B—C11B—C19B	112.55 (18)	C14D—C13D—C12D	124.4 (3)
N1B—C11B—C12B	111.06 (19)	C18D—C13D—C12D	118.3 (3)
C19B—C11B—C12B	110.36 (18)	C13D—C14D—C15D	120.1 (4)
N1B—C11B—H11B	107.5	C13D—C14D—H14D	119.9
C19B—C11B—H11B	107.5	C15D—C14D—H14D	119.9
C12B—C11B—H11B	107.5	C16D—C15D—C14D	121.1 (4)
C13B—C12B—C11B	114.4 (2)	C16D—C15D—H15D	119.5
C13B—C12B—H12C	108.7	C14D—C15D—H15D	119.5
C11B—C12B—H12C	108.7	C17D—C16D—C15D	118.8 (3)
C13B—C12B—H12D	108.7	C17D—C16D—H16D	120.6
C11B—C12B—H12D	108.7	C15D—C16D—H16D	120.6
H12C—C12B—H12D	107.6	C16D—C17D—C18D	120.6 (4)
C18B—C13B—C14B	118.6 (3)	C16D—C17D—H17D	119.7
C18B—C13B—C12B	120.7 (2)	C18D—C17D—H17D	119.7
C14B—C13B—C12B	120.7 (3)	C13D—C18D—C17D	121.9 (3)
C15B—C14B—C13B	120.7 (4)	C13D—C18D—H18D	119
C15B—C14B—H14B	119.7	C17D—C18D—H18D	119
C13B—C14B—H14B	119.7	C21D—C20D—C19D	114.4 (2)
C16B—C15B—C14B	120.1 (4)	C21D—C20D—H20G	108.6
C16B—C15B—H15B	120	C19D—C20D—H20G	108.6
C14B—C15B—H15B	120	C21D—C20D—H20H	108.6
C17B—C16B—C15B	119.9 (3)	C19D—C20D—H20H	108.6
C17B—C16B—H16B	120.1	H20G—C20D—H20H	107.6
C15B—C16B—H16B	120.1	N2D—C21D—C20D	109.77 (19)
C16B—C17B—C18B	120.5 (4)	N2D—C21D—C22D	112.1 (2)
C16B—C17B—H17B	119.7	C20D—C21D—C22D	110.0 (2)
C18B—C17B—H17B	119.7	N2D—C21D—H21D	108.3
C13B—C18B—C17B	120.2 (3)	C20D—C21D—H21D	108.3
C13B—C18B—H18B	119.9	C22D—C21D—H21D	108.3
C17B—C18B—H18B	119.9	C23D—C22D—C21D	113.9 (2)
O3B—C19B—C20B	111.11 (18)	C23D—C22D—H22G	108.8
O3B—C19B—C11B	112.86 (18)	C21D—C22D—H22G	108.8
C20B—C19B—C11B	113.36 (18)	C23D—C22D—H22H	108.8
O3B—C19B—H19B	106.3	C21D—C22D—H22H	108.8
C20B—C19B—H19B	106.3	H22G—C22D—H22H	107.7
C11B—C19B—H19B	106.3	C28D—C23D—C24D	118.3 (2)
C19B—C20B—C21B	112.31 (18)	C28D—C23D—C22D	122.4 (2)
C19B—C20B—H20C	109.1	C24D—C23D—C22D	119.3 (2)
C21B—C20B—H20C	109.1	C25D—C24D—C23D	121.2 (2)
C19B—C20B—H20D	109.1	C25D—C24D—H24D	119.4
C21B—C20B—H20D	109.1	C23D—C24D—H24D	119.4
H20C—C20B—H20D	107.9	C26D—C25D—C24D	119.7 (2)
N2B—C21B—C20B	111.16 (18)	C26D—C25D—H25D	120.2
N2B—C21B—C22B	108.06 (18)	C24D—C25D—H25D	120.2
C20B—C21B—C22B	112.68 (18)	C27D—C26D—C25D	120.0 (2)
N2B—C21B—H21B	108.3	C27D—C26D—H26D	120
C20B—C21B—H21B	108.3	C25D—C26D—H26D	120
C22B—C21B—H21B	108.3	C26D—C27D—C28D	120.3 (3)
C23B—C22B—C21B	112.74 (18)	C26D—C27D—H27D	119.8
C23B—C22B—H22C	109	C28D—C27D—H27D	119.8
C21B—C22B—H22C	109	C23D—C28D—C27D	120.4 (2)
C23B—C22B—H22D	109	C23D—C28D—H28D	119.8
C21B—C22B—H22D	109	C27D—C28D—H28D	119.8
H22C—C22B—H22D	107.8	O4D—C29D—N2D	123.9 (2)
C24B—C23B—C28B	118.6 (2)	O4D—C29D—C30D	121.7 (2)
C24B—C23B—C22B	121.6 (2)	N2D—C29D—C30D	114.4 (2)
C28B—C23B—C22B	119.8 (2)	N3D—C30D—C31D	114.3 (2)
C25B—C24B—C23B	120.4 (2)	N3D—C30D—C29D	108.28 (19)
C25B—C24B—H24B	119.8	C31D—C30D—C29D	110.7 (2)
C23B—C24B—H24B	119.8	N3D—C30D—H30D	107.8
C26B—C25B—C24B	120.5 (2)	C31D—C30D—H30D	107.8
C26B—C25B—H25B	119.8	C29D—C30D—H30D	107.8
C24B—C25B—H25B	119.8	C32D—C31D—C30D	108.7 (2)
C25B—C26B—C27B	119.6 (2)	C32D—C31D—C33D	110.7 (3)
C25B—C26B—H26B	120.2	C30D—C31D—C33D	110.7 (2)
C27B—C26B—H26B	120.2	C32D—C31D—H31D	108.9
C28B—C27B—C26B	119.8 (2)	C30D—C31D—H31D	108.9
C28B—C27B—H27B	120.1	C33D—C31D—H31D	108.9
C26B—C27B—H27B	120.1	C31D—C32D—H32J	109.5
C27B—C28B—C23B	121.1 (2)	C31D—C32D—H32K	109.5
C27B—C28B—H28B	119.4	H32J—C32D—H32K	109.5
C23B—C28B—H28B	119.4	C31D—C32D—H32L	109.5
O4B—C29B—N2B	123.8 (2)	H32J—C32D—H32L	109.5
O4B—C29B—C30B	120.8 (2)	H32K—C32D—H32L	109.5
N2B—C29B—C30B	115.35 (19)	C31D—C33D—H33J	109.5
N3B—C30B—C29B	108.70 (18)	C31D—C33D—H33K	109.5
N3B—C30B—C31B	113.13 (18)	H33J—C33D—H33K	109.5
C29B—C30B—C31B	111.54 (18)	C31D—C33D—H33L	109.5
N3B—C30B—H30B	107.8	H33J—C33D—H33L	109.5
C29B—C30B—H30B	107.8	H33K—C33D—H33L	109.5
C31B—C30B—H30B	107.8	O5D—C34D—N3D	120.9 (2)
C33B—C31B—C32B	110.1 (2)	O5D—C34D—N4D	120.7 (3)
C33B—C31B—C30B	111.38 (19)	N3D—C34D—N4D	118.4 (3)
C32B—C31B—C30B	107.9 (2)	N3D—C35D—C36D	111.7 (3)
C33B—C31B—H31B	109.1	N3D—C35D—H35G	109.3
C32B—C31B—H31B	109.1	C36D—C35D—H35G	109.3
C30B—C31B—H31B	109.1	N3D—C35D—H35H	109.3
C31B—C32B—H32D	109.5	C36D—C35D—H35H	109.3
C31B—C32B—H32E	109.5	H35G—C35D—H35H	107.9
H32D—C32B—H32E	109.5	C35D—C36D—C37D	110.7 (3)
C31B—C32B—H32F	109.5	C35D—C36D—H36G	109.5
H32D—C32B—H32F	109.5	C37D—C36D—H36G	109.5
H32E—C32B—H32F	109.5	C35D—C36D—H36H	109.5
C31B—C33B—H33D	109.5	C37D—C36D—H36H	109.5
C31B—C33B—H33E	109.5	H36G—C36D—H36H	108.1
H33D—C33B—H33E	109.5	N4D—C37D—C36D	109.8 (3)
C31B—C33B—H33F	109.5	N4D—C37D—H37G	109.7
H33D—C33B—H33F	109.5	C36D—C37D—H37G	109.7
H33E—C33B—H33F	109.5	N4D—C37D—H37H	109.7
O5B—C34B—N3B	122.8 (2)	C36D—C37D—H37H	109.7
O5B—C34B—N4B	118.6 (2)	H37G—C37D—H37H	108.2
N3B—C34B—N4B	118.6 (2)	C10D—N1D—C11D	121.7 (2)
N3B—C35B—C36B	110.9 (2)	C10D—N1D—H1D	119.2
N3B—C35B—H35C	109.5	C11D—N1D—H1D	119.2
C36B—C35B—H35C	109.5	C29D—N2D—C21D	124.7 (2)
N3B—C35B—H35D	109.5	C29D—N2D—H2D	117.7
C36B—C35B—H35D	109.5	C21D—N2D—H2D	117.7
H35C—C35B—H35D	108	C34D—N3D—C35D	121.7 (2)
C37B—C36B—C35B	109.9 (2)	C34D—N3D—C30D	116.5 (2)
C37B—C36B—H36C	109.7	C35D—N3D—C30D	119.6 (2)
C35B—C36B—H36C	109.7	C34D—N4D—C37D	123.5 (3)
C37B—C36B—H36D	109.7	C34D—N4D—H4D	118.2
C35B—C36B—H36D	109.7	C37D—N4D—H4D	118.2
H36C—C36B—H36D	108.2	C1D—O1D—C9D	110.82 (18)
N4B—C37B—C36B	108.9 (2)	C19D—O3D—H3D	109.5
N4B—C37B—H37C	109.9	O1—C1—C2	114.1 (3)
C36B—C37B—H37C	109.9	O1—C1—H1E	108.7
N4B—C37B—H37D	109.9	C2—C1—H1E	108.7
C36B—C37B—H37D	109.9	O1—C1—H1F	108.7
H37C—C37B—H37D	108.3	C2—C1—H1F	108.7
C10B—N1B—C11B	121.51 (19)	H1E—C1—H1F	107.6
C10B—N1B—H1B	119.2	O2—C2—C1	112.9 (3)
C11B—N1B—H1B	119.2	O2—C2—H2E	109
C29B—N2B—C21B	122.70 (19)	C1—C2—H2E	109
C29B—N2B—H2B	118.7	O2—C2—H2F	109
C21B—N2B—H2B	118.6	C1—C2—H2F	109
C34B—N3B—C30B	120.26 (19)	H2E—C2—H2F	107.8
C34B—N3B—C35B	120.65 (19)	C1—O1—H1	109.5
C30B—N3B—C35B	119.04 (18)	C2—O2—H2	109.5
C34B—N4B—C37B	126.2 (2)	C3—O3—H3O	109.5
C34B—N4B—H4B	116.9	O3—C3—C3'	113.8 (6)
C37B—N4B—H4B	116.9	O3—C3—H31	108.8
C1B—O1B—C9B	114.90 (17)	C3'—C3—H31	108.8
C19B—O3B—H3B	109.5	O3—C3—H32	108.8
O1C—C1C—C2C	119.1 (2)	C3'—C3—H32	108.8
O1C—C1C—C6C	118.2 (2)	H31—C3—H32	107.7
C2C—C1C—C6C	122.6 (3)	O3'—C3'—C3	109.2 (7)
C3C—C2C—C1C	117.5 (2)	O3'—C3'—H31'	109.8
C3C—C2C—C7C	120.2 (2)	C3—C3'—H31'	109.8
C1C—C2C—C7C	122.3 (3)	O3'—C3'—H32'	109.8
C4C—C3C—C2C	121.0 (3)	C3—C3'—H32'	109.8
C4C—C3C—H3CC	119.5	H31'—C3'—H32'	108.3
C2C—C3C—H3CC	119.5	C3'—O3'—H3O'	109.5
C3C—C4C—C5C	120.4 (3)	H1WA—O1W—H1WB	104.5
C3C—C4C—H4CC	119.8	H2WA—O2W—H2WB	104.5
C5C—C4C—H4CC	119.8	H3WA—O3W—H3WB	104.5
C4C—C5C—C6C	121.1 (3)	H4WA—O4W—H4WB	104.5
C4C—C5C—H5C	119.5	H4WA—O4W—H4WA^i^	105.6
C6C—C5C—H5C	119.5	H4WB—O4W—H4WA^i^	21.4
C5C—C6C—C1C	117.4 (3)	H4WA—O4W—H4WB^i^	21.4
C5C—C6C—C8C	122.3 (3)	H4WB—O4W—H4WB^i^	111.5
C1C—C6C—C8C	120.2 (3)	H4WA^i^—O4W—H4WB^i^	104.5
C2C—C7C—H7CA	109.5		
			
C6A—C1A—C2A—C3A	0.8 (4)	C6C—C1C—C2C—C3C	−3.5 (4)
O1A—C1A—C2A—C3A	176.8 (2)	O1C—C1C—C2C—C7C	−1.0 (4)
C6A—C1A—C2A—C7A	−178.3 (2)	C6C—C1C—C2C—C7C	174.9 (2)
O1A—C1A—C2A—C7A	−2.2 (3)	C1C—C2C—C3C—C4C	1.5 (4)
C1A—C2A—C3A—C4A	−0.5 (4)	C7C—C2C—C3C—C4C	−176.9 (3)
C7A—C2A—C3A—C4A	178.6 (3)	C2C—C3C—C4C—C5C	1.3 (4)
C2A—C3A—C4A—C5A	−0.1 (4)	C3C—C4C—C5C—C6C	−2.2 (4)
C3A—C4A—C5A—C6A	0.4 (4)	C4C—C5C—C6C—C1C	0.3 (4)
C4A—C5A—C6A—C1A	−0.1 (4)	C4C—C5C—C6C—C8C	179.9 (3)
C4A—C5A—C6A—C8A	−177.9 (3)	O1C—C1C—C6C—C5C	178.5 (2)
C2A—C1A—C6A—C5A	−0.5 (4)	C2C—C1C—C6C—C5C	2.6 (4)
O1A—C1A—C6A—C5A	−176.6 (2)	O1C—C1C—C6C—C8C	−1.2 (4)
C2A—C1A—C6A—C8A	177.3 (3)	C2C—C1C—C6C—C8C	−177.0 (2)
O1A—C1A—C6A—C8A	1.3 (4)	O1C—C9C—C10C—O2C	−171.9 (3)
O1A—C9A—C10A—O2A	−175.0 (2)	O1C—C9C—C10C—N1C	6.7 (4)
O1A—C9A—C10A—N1A	5.1 (3)	N1C—C11C—C12C—C13C	−71.8 (3)
N1A—C11A—C12A—C13A	−56.9 (3)	C19C—C11C—C12C—C13C	164.9 (2)
C19A—C11A—C12A—C13A	177.6 (2)	C11C—C12C—C13C—C14C	−1.1 (4)
C11A—C12A—C13A—C18A	89.4 (3)	C11C—C12C—C13C—C18C	−179.1 (3)
C11A—C12A—C13A—C14A	−88.9 (3)	C18C—C13C—C14C—C15C	−0.9 (5)
C18A—C13A—C14A—C15A	−0.2 (4)	C12C—C13C—C14C—C15C	−178.9 (3)
C12A—C13A—C14A—C15A	178.1 (3)	C13C—C14C—C15C—C16C	0.8 (6)
C13A—C14A—C15A—C16A	−1.0 (5)	C14C—C15C—C16C—C17C	−0.6 (6)
C14A—C15A—C16A—C17A	0.9 (5)	C15C—C16C—C17C—C18C	0.7 (5)
C15A—C16A—C17A—C18A	0.4 (5)	C14C—C13C—C18C—C17C	1.0 (5)
C14A—C13A—C18A—C17A	1.5 (4)	C12C—C13C—C18C—C17C	179.1 (3)
C12A—C13A—C18A—C17A	−176.9 (3)	C16C—C17C—C18C—C13C	−0.9 (5)
C16A—C17A—C18A—C13A	−1.6 (4)	N1C—C11C—C19C—O3C	173.4 (2)
N1A—C11A—C19A—O3A	−76.6 (2)	C12C—C11C—C19C—O3C	−62.3 (3)
C12A—C11A—C19A—O3A	47.7 (2)	N1C—C11C—C19C—C20C	−63.6 (3)
N1A—C11A—C19A—C20A	50.2 (3)	C12C—C11C—C19C—C20C	60.7 (3)
C12A—C11A—C19A—C20A	174.41 (19)	O3C—C19C—C20C—C21C	−123.0 (3)
O3A—C19A—C20A—C21A	−54.5 (2)	C11C—C19C—C20C—C21C	116.7 (3)
C11A—C19A—C20A—C21A	177.43 (18)	C19C—C20C—C21C—N2C	56.3 (3)
C19A—C20A—C21A—N2A	170.88 (18)	C19C—C20C—C21C—C22C	178.5 (2)
C19A—C20A—C21A—C22A	−67.4 (2)	N2C—C21C—C22C—C23C	−61.0 (3)
N2A—C21A—C22A—C23A	−62.1 (2)	C20C—C21C—C22C—C23C	177.7 (2)
C20A—C21A—C22A—C23A	175.38 (19)	C21C—C22C—C23C—C28C	90.2 (3)
C21A—C22A—C23A—C24A	−60.7 (3)	C21C—C22C—C23C—C24C	−89.8 (3)
C21A—C22A—C23A—C28A	121.6 (2)	C28C—C23C—C24C—C25C	0.6 (4)
C28A—C23A—C24A—C25A	1.6 (4)	C22C—C23C—C24C—C25C	−179.3 (2)
C22A—C23A—C24A—C25A	−176.2 (2)	C23C—C24C—C25C—C26C	−0.4 (4)
C23A—C24A—C25A—C26A	−0.1 (4)	C24C—C25C—C26C—C27C	−0.1 (4)
C24A—C25A—C26A—C27A	−1.3 (4)	C25C—C26C—C27C—C28C	0.4 (4)
C25A—C26A—C27A—C28A	1.2 (4)	C26C—C27C—C28C—C23C	−0.2 (4)
C26A—C27A—C28A—C23A	0.4 (4)	C24C—C23C—C28C—C27C	−0.3 (4)
C24A—C23A—C28A—C27A	−1.7 (4)	C22C—C23C—C28C—C27C	179.6 (2)
C22A—C23A—C28A—C27A	176.0 (2)	O4C—C29C—C30C—N3C	−74.6 (3)
O4A—C29A—C30A—N3A	−78.2 (3)	N2C—C29C—C30C—N3C	105.1 (3)
N2A—C29A—C30A—N3A	100.0 (2)	O4C—C29C—C30C—C31C	51.3 (3)
O4A—C29A—C30A—C31A	45.5 (3)	N2C—C29C—C30C—C31C	−128.9 (2)
N2A—C29A—C30A—C31A	−136.3 (2)	N3C—C30C—C31C—C33C	−58.2 (3)
N3A—C30A—C31A—C33A	−63.4 (3)	C29C—C30C—C31C—C33C	178.1 (2)
C29A—C30A—C31A—C33A	174.9 (2)	N3C—C30C—C31C—C32C	179.5 (3)
N3A—C30A—C31A—C32A	173.1 (2)	C29C—C30C—C31C—C32C	55.8 (3)
C29A—C30A—C31A—C32A	51.4 (3)	N3C—C35C—C36C—C37C	45.7 (6)
N3A—C35A—C36A—C37A	−53.6 (3)	C35C—C36C—C37C—N4C	−36.7 (6)
C35A—C36A—C37A—N4A	47.4 (3)	O2C—C10C—N1C—C11C	1.3 (5)
O2A—C10A—N1A—C11A	5.9 (3)	C9C—C10C—N1C—C11C	−177.2 (3)
C9A—C10A—N1A—C11A	−174.2 (2)	C12C—C11C—N1C—C10C	144.6 (3)
C19A—C11A—N1A—C10A	−133.1 (2)	C19C—C11C—N1C—C10C	−89.4 (3)
C12A—C11A—N1A—C10A	102.4 (2)	O4C—C29C—N2C—C21C	−4.5 (4)
O4A—C29A—N2A—C21A	8.4 (3)	C30C—C29C—N2C—C21C	175.8 (2)
C30A—C29A—N2A—C21A	−169.79 (18)	C20C—C21C—N2C—C29C	−130.4 (3)
C20A—C21A—N2A—C29A	−116.9 (2)	C22C—C21C—N2C—C29C	108.7 (3)
C22A—C21A—N2A—C29A	119.3 (2)	O5C—C34C—N3C—C35C	−168.5 (3)
O5A—C34A—N3A—C30A	−1.3 (3)	N4C—C34C—N3C—C35C	12.5 (4)
N4A—C34A—N3A—C30A	179.6 (2)	O5C—C34C—N3C—C30C	−4.2 (4)
O5A—C34A—N3A—C35A	176.8 (2)	N4C—C34C—N3C—C30C	176.9 (3)
N4A—C34A—N3A—C35A	−2.3 (3)	C36C—C35C—N3C—C34C	−32.9 (5)
C29A—C30A—N3A—C34A	−108.2 (2)	C36C—C35C—N3C—C30C	163.0 (3)
C31A—C30A—N3A—C34A	127.4 (2)	C29C—C30C—N3C—C34C	−95.9 (3)
C29A—C30A—N3A—C35A	73.7 (2)	C31C—C30C—N3C—C34C	139.4 (3)
C31A—C30A—N3A—C35A	−50.7 (3)	C29C—C30C—N3C—C35C	68.4 (4)
C36A—C35A—N3A—C34A	31.8 (3)	C31C—C30C—N3C—C35C	−56.2 (4)
C36A—C35A—N3A—C30A	−150.1 (2)	O5C—C34C—N4C—C37C	177.5 (4)
O5A—C34A—N4A—C37A	176.8 (2)	N3C—C34C—N4C—C37C	−3.5 (5)
N3A—C34A—N4A—C37A	−4.1 (4)	C36C—C37C—N4C—C34C	15.5 (6)
C36A—C37A—N4A—C34A	−19.5 (4)	C2C—C1C—O1C—C9C	−87.0 (3)
C2A—C1A—O1A—C9A	90.0 (2)	C6C—C1C—O1C—C9C	97.0 (3)
C6A—C1A—O1A—C9A	−93.7 (2)	C10C—C9C—O1C—C1C	176.1 (2)
C10A—C9A—O1A—C1A	−148.74 (19)	C6D—C1D—C2D—C3D	3.2 (4)
C6B—C1B—C2B—C3B	1.8 (3)	O1D—C1D—C2D—C3D	−178.0 (2)
O1B—C1B—C2B—C3B	−175.2 (2)	C6D—C1D—C2D—C7D	−175.9 (2)
C6B—C1B—C2B—C7B	−177.5 (2)	O1D—C1D—C2D—C7D	2.9 (3)
O1B—C1B—C2B—C7B	5.5 (3)	C1D—C2D—C3D—C4D	0.0 (4)
C1B—C2B—C3B—C4B	−0.9 (4)	C7D—C2D—C3D—C4D	179.1 (2)
C7B—C2B—C3B—C4B	178.4 (3)	C2D—C3D—C4D—C5D	−2.4 (4)
C2B—C3B—C4B—C5B	−0.5 (4)	C3D—C4D—C5D—C6D	1.7 (4)
C3B—C4B—C5B—C6B	1.0 (4)	C4D—C5D—C6D—C1D	1.3 (4)
C4B—C5B—C6B—C1B	0.0 (4)	C4D—C5D—C6D—C8D	−177.2 (3)
C4B—C5B—C6B—C8B	−179.5 (2)	C2D—C1D—C6D—C5D	−3.9 (4)
C2B—C1B—C6B—C5B	−1.4 (3)	O1D—C1D—C6D—C5D	177.3 (2)
O1B—C1B—C6B—C5B	175.7 (2)	C2D—C1D—C6D—C8D	174.6 (2)
C2B—C1B—C6B—C8B	178.1 (2)	O1D—C1D—C6D—C8D	−4.2 (3)
O1B—C1B—C6B—C8B	−4.8 (3)	O1D—C9D—C10D—O2D	−169.3 (2)
O1B—C9B—C10B—O2B	−174.9 (2)	O1D—C9D—C10D—N1D	10.2 (3)
O1B—C9B—C10B—N1B	5.7 (3)	O3D—C19D—C11D—N1D	169.0 (2)
N1B—C11B—C12B—C13B	−60.3 (3)	C20D—C19D—C11D—N1D	−66.6 (3)
C19B—C11B—C12B—C13B	174.2 (2)	O3D—C19D—C11D—C12D	−67.0 (3)
C11B—C12B—C13B—C18B	87.0 (3)	C20D—C19D—C11D—C12D	57.3 (3)
C11B—C12B—C13B—C14B	−92.9 (3)	N1D—C11D—C12D—C13D	−61.9 (3)
C18B—C13B—C14B—C15B	−0.3 (4)	C19D—C11D—C12D—C13D	175.5 (2)
C12B—C13B—C14B—C15B	179.6 (3)	C11D—C12D—C13D—C14D	−7.4 (4)
C13B—C14B—C15B—C16B	−0.5 (6)	C11D—C12D—C13D—C18D	170.1 (2)
C14B—C15B—C16B—C17B	0.9 (6)	C18D—C13D—C14D—C15D	1.0 (5)
C15B—C16B—C17B—C18B	−0.5 (6)	C12D—C13D—C14D—C15D	178.6 (3)
C14B—C13B—C18B—C17B	0.6 (4)	C13D—C14D—C15D—C16D	2.1 (7)
C12B—C13B—C18B—C17B	−179.2 (3)	C14D—C15D—C16D—C17D	−3.8 (7)
C16B—C17B—C18B—C13B	−0.2 (5)	C15D—C16D—C17D—C18D	2.3 (6)
N1B—C11B—C19B—O3B	−76.6 (2)	C14D—C13D—C18D—C17D	−2.5 (5)
C12B—C11B—C19B—O3B	48.1 (3)	C12D—C13D—C18D—C17D	179.8 (3)
N1B—C11B—C19B—C20B	50.8 (3)	C16D—C17D—C18D—C13D	0.9 (5)
C12B—C11B—C19B—C20B	175.51 (19)	O3D—C19D—C20D—C21D	−109.2 (2)
O3B—C19B—C20B—C21B	−54.4 (2)	C11D—C19D—C20D—C21D	130.3 (2)
C11B—C19B—C20B—C21B	177.31 (19)	C19D—C20D—C21D—N2D	60.3 (3)
C19B—C20B—C21B—N2B	170.79 (18)	C19D—C20D—C21D—C22D	−175.9 (2)
C19B—C20B—C21B—C22B	−67.7 (2)	N2D—C21D—C22D—C23D	−77.9 (3)
N2B—C21B—C22B—C23B	−62.6 (2)	C20D—C21D—C22D—C23D	159.7 (2)
C20B—C21B—C22B—C23B	174.14 (19)	C21D—C22D—C23D—C28D	96.2 (3)
C21B—C22B—C23B—C24B	112.8 (2)	C21D—C22D—C23D—C24D	−83.2 (3)
C21B—C22B—C23B—C28B	−67.7 (3)	C28D—C23D—C24D—C25D	0.6 (4)
C28B—C23B—C24B—C25B	−1.7 (3)	C22D—C23D—C24D—C25D	180.0 (2)
C22B—C23B—C24B—C25B	177.8 (2)	C23D—C24D—C25D—C26D	0.4 (4)
C23B—C24B—C25B—C26B	1.1 (4)	C24D—C25D—C26D—C27D	−1.0 (4)
C24B—C25B—C26B—C27B	−0.1 (4)	C25D—C26D—C27D—C28D	0.6 (4)
C25B—C26B—C27B—C28B	−0.2 (4)	C24D—C23D—C28D—C27D	−1.0 (4)
C26B—C27B—C28B—C23B	−0.4 (4)	C22D—C23D—C28D—C27D	179.6 (2)
C24B—C23B—C28B—C27B	1.4 (3)	C26D—C27D—C28D—C23D	0.4 (4)
C22B—C23B—C28B—C27B	−178.1 (2)	O4D—C29D—C30D—N3D	−73.1 (3)
O4B—C29B—C30B—N3B	−81.2 (3)	N2D—C29D—C30D—N3D	106.1 (2)
N2B—C29B—C30B—N3B	97.1 (2)	O4D—C29D—C30D—C31D	52.9 (3)
O4B—C29B—C30B—C31B	44.2 (3)	N2D—C29D—C30D—C31D	−127.9 (2)
N2B—C29B—C30B—C31B	−137.5 (2)	N3D—C30D—C31D—C32D	−179.4 (2)
N3B—C30B—C31B—C33B	−51.1 (3)	C29D—C30D—C31D—C32D	58.0 (3)
C29B—C30B—C31B—C33B	−174.07 (19)	N3D—C30D—C31D—C33D	−57.7 (3)
N3B—C30B—C31B—C32B	−172.2 (2)	C29D—C30D—C31D—C33D	179.8 (2)
C29B—C30B—C31B—C32B	64.9 (3)	N3D—C35D—C36D—C37D	−50.3 (4)
N3B—C35B—C36B—C37B	55.4 (3)	C35D—C36D—C37D—N4D	52.5 (4)
C35B—C36B—C37B—N4B	−49.8 (3)	O2D—C10D—N1D—C11D	2.2 (4)
O2B—C10B—N1B—C11B	5.4 (3)	C9D—C10D—N1D—C11D	−177.1 (2)
C9B—C10B—N1B—C11B	−175.3 (2)	C12D—C11D—N1D—C10D	144.9 (2)
C19B—C11B—N1B—C10B	−135.7 (2)	C19D—C11D—N1D—C10D	−90.2 (3)
C12B—C11B—N1B—C10B	100.0 (2)	O4D—C29D—N2D—C21D	−0.4 (4)
O4B—C29B—N2B—C21B	12.2 (3)	C30D—C29D—N2D—C21D	−179.6 (2)
C30B—C29B—N2B—C21B	−166.02 (18)	C20D—C21D—N2D—C29D	−141.4 (2)
C20B—C21B—N2B—C29B	−113.7 (2)	C22D—C21D—N2D—C29D	96.0 (3)
C22B—C21B—N2B—C29B	122.2 (2)	O5D—C34D—N3D—C35D	−167.7 (3)
O5B—C34B—N3B—C30B	1.8 (3)	N4D—C34D—N3D—C35D	12.6 (4)
N4B—C34B—N3B—C30B	−177.6 (2)	O5D—C34D—N3D—C30D	−4.7 (4)
O5B—C34B—N3B—C35B	−175.5 (2)	N4D—C34D—N3D—C30D	175.6 (2)
N4B—C34B—N3B—C35B	5.1 (3)	C36D—C35D—N3D—C34D	18.0 (4)
C29B—C30B—N3B—C34B	−117.8 (2)	C36D—C35D—N3D—C30D	−144.5 (3)
C31B—C30B—N3B—C34B	117.7 (2)	C31D—C30D—N3D—C34D	152.7 (2)
C29B—C30B—N3B—C35B	59.5 (3)	C29D—C30D—N3D—C34D	−83.4 (3)
C31B—C30B—N3B—C35B	−65.0 (3)	C31D—C30D—N3D—C35D	−43.9 (3)
C36B—C35B—N3B—C34B	−33.0 (3)	C29D—C30D—N3D—C35D	80.0 (3)
C36B—C35B—N3B—C30B	149.6 (2)	O5D—C34D—N4D—C37D	170.9 (3)
O5B—C34B—N4B—C37B	−180.0 (2)	N3D—C34D—N4D—C37D	−9.4 (5)
N3B—C34B—N4B—C37B	−0.5 (3)	C36D—C37D—N4D—C34D	−23.6 (5)
C36B—C37B—N4B—C34B	24.1 (3)	C6D—C1D—O1D—C9D	−91.8 (3)
C2B—C1B—O1B—C9B	−82.0 (3)	C2D—C1D—O1D—C9D	89.3 (3)
C6B—C1B—O1B—C9B	100.8 (2)	C10D—C9D—O1D—C1D	178.3 (2)
C10B—C9B—O1B—C1B	−149.6 (2)	O1—C1—C2—O2	68.4 (4)
O1C—C1C—C2C—C3C	−179.3 (2)	O3—C3—C3'—O3'	−176.5 (8)

Symmetry code: (i) −*x*+1, *y*, −*z*+2.

### (2S)-N-[(2S,4S,5S)-5-[2-(2,6-Dimethylphenoxy)acetamido]-4-hydroxy-1,6-diphenylhexan-2-yl]-3-methyl-2-(2-oxo-1,3-diazinan-1-yl)butanamide–ethane-1,2-diol–water (8/2/1/7) Hydrogen-bond geometry (Å, º)

**Table:**

*D*—H···*A*	*D*—H	H···*A*	*D*···*A*	*D*—H···*A*
N1*A*—H1*A*···O5*B*	0.88	2.18	2.997 (2)	154
N2*A*—H2*A*···O1*W*^ii^	0.88	2.06	2.906 (3)	160
N4*A*—H4*A*···O1*B*^ii^	0.88	2.08	2.933 (3)	163
O3*A*—H3*A*···O5*B*	0.84	1.91	2.750 (2)	173
N1*B*—H1*B*···O5*A*^iii^	0.88	2.2	3.005 (2)	152
N2*B*—H2*B*···O2*W*	0.88	2.05	2.886 (3)	159
N4*B*—H4*B*···O1*A*	0.88	2.08	2.932 (3)	164
O3*B*—H3*B*···O5*A*^iii^	0.84	1.94	2.771 (2)	170
N1*C*—H1*C*···O2*B*	0.88	2.21	3.027 (3)	155
N2*C*—H2*C*···O1	0.88	2.18	2.978 (3)	151
N4*C*—H4*C*···O3*W*	0.88	2.19	2.983 (3)	150
O3*C*—H3*C*···O1	0.81	2.08	2.869 (4)	165
N1*D*—H1*D*···O2*A*^iv^	0.88	2.23	3.058 (3)	156
N2*D*—H2*D*···O2	0.88	2.13	2.973 (3)	159
N4*D*—H4*D*···O2*C*^v^	0.88	2.18	2.825 (3)	130
O3*D*—H3*D*···O2	0.84	1.89	2.730 (3)	178
O1—H1···O5*D*	0.84	1.85	2.606 (3)	149
O2—H2···O5*C*	0.84	1.74	2.581 (3)	174
O3—H3*O*···O3*W*	0.84	2.03	2.856 (12)	167
O1*W*—H1*WB*···O5*A*^iii^	0.87	2.05	2.861 (2)	154
O1*W*—H1*WA*···O4*B*	0.87	1.96	2.818 (2)	169
O2*W*—H2*WA*···O4*A*	0.87	1.95	2.810 (2)	171
O2*W*—H2*WB*···O5*B*	0.87	2.08	2.907 (2)	158
O3*W*—H3*WA*···O2*D*^i^	0.87	1.9	2.769 (3)	173
O3*W*—H3*WB*···O4*W*	0.87	1.93	2.798 (2)	172
O4*W*—H4*WB*···O3*D*	0.87	1.89	2.736 (3)	164
O4*W*—H4*WA*···O3*D*^i^	0.87	1.89	2.736 (3)	164
O1—H1···O5*D*	0.84	1.85	2.606 (3)	149
O2—H2···O5*C*	0.84	1.74	2.581 (3)	174
O3—H3*O*···O3*W*	0.84	2.03	2.856 (12)	167

Symmetry codes: (i) −*x*+1, *y*, −*z*+2; (ii) *x*, *y*+1, *z*; (iii) *x*, *y*−1, *z*; (iv) *x*−1/2, *y*−1/2, *z*; (v) −*x*+1, *y*, −*z*+1.
